# Three New Ionone Glycosides from *Rhododendron capitatum* Maxim

**DOI:** 10.3390/molecules29112462

**Published:** 2024-05-23

**Authors:** Jun-Ren Yang, Yue-Tong Zhu, Yi-Qin Zeng, Hong-Quan Li, Chun-Huan Li, Jin-Ming Gao

**Affiliations:** 1 Shaanxi Key Laboratory of Natural Products & Chemical Biology, College of Chemistry & Pharmacy, Northwest A&F University, Yangling 712100, China; yang@jiaherb.com (J.-R.Y.); 15037143303@163.com (Y.-T.Z.); zengyiqin0619@163.com (Y.-Q.Z.); 18193816925@163.com (H.-Q.L.); 2 Shaanxi Jiahe Phytochemistry Company, Xi’an 710077, China

**Keywords:** *Rhododendron capitatum*, sesquiterpenoids, ionone glycosides, anti-inflammatory activity

## Abstract

Six ionone glycosides (**1**–**3** and **5**–**7**), including three new ones, named capitsesqsides A−C (**1**–**3**), together with an eudesmane sesquiterpenoid glycoside (**4**) and three known triterpenoid saponins (**8**–**10**) were isolated from *Rhododendron capitatum*. The structures of these compounds were determined by extensive spectroscopic techniques (MS, UV, 1D-NMR, and 2D-NMR) and comparison with data reported in the literature. The absolute configurations were determined by comparison of the experimental and theoretically calculated ECD curves and LC-MS analyses after acid hydrolysis and derivatization. The anti-inflammatory activities of these compounds were evaluated in the LPS-induced RAW264.7 cells. Molecular docking demonstrated that **2** has a favorable affinity for NLRP3 and iNOS.

## 1. Introduction

Ionones are decarbonated sesquiterpenes comprising cyclised isoprene units. They are widely distributed in plants of the Scrophulariaceae [1], Solanaceae [2], and Simaroubaceae [3] families, with a varied range of pharmacological activities including *α*-glucosidase inhibition [1] and anti-inflammatory [3,4,5], anti-tumour [5], and anti-platelet aggregation properties [6].

**Figure 1:**
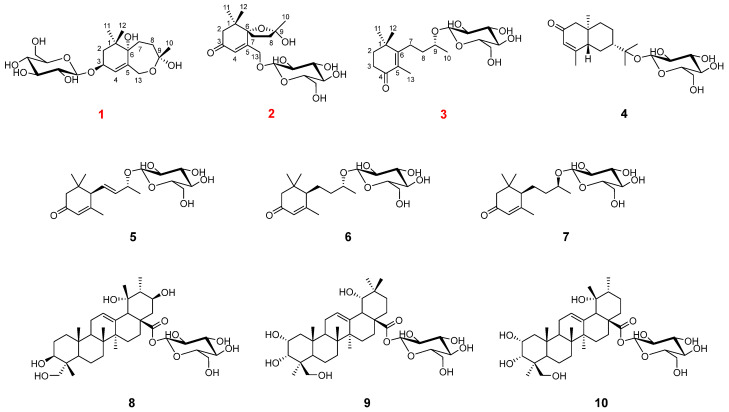
Structures of compounds **1**–**10** (**1**–**3** were new compounds).

*Rhododendron capitatum* Maxim belongs to the Ericaceae family and is a small deciduous shrub, mainly distributed in the Shaanxi and Qinghai Provinces of China [7]. *R. capitatum* has a high horticultural value due to its bright colours and beautiful flowers and is often grown as an ornamental. As a Tibetan medicine for the treatment of gastric cold, abdominal pain, pharyngalgia, cough, and inflammation [8], previous phytochemical research has shown it contains a variety of structurally diverse meroterpenoids, grayanane diterpenoids, flavonoids, and coumarins [9]. These compounds exhibit a variety of biological and pharmacological activities, including anti-inflammatory, antiviral, cytotoxic, and hypoglycaemic activities [7,8,9,10]. As part of our continuing studies on chemical components with novel structures and significant pharmacological activities from folk medicinal plants found in the Qinling region [11,12], chemical investigations of the aerial parts of *R. capitatum* were undertaken, leading to the isolation and identification of three new ionone glycosides (**1**–**3**) and seven known compounds (**4**–**10**). This is the first report on the sesquiterpenoid compounds from *R. capitatum* (Figure 1), wherein compound **1** was a novel ionone with a 6/7 bicyclic skeleton. All the isolated compounds were evaluated for anti-inflammatory activities in the LPS-induced RAW264.7 cells model.

## 2. Results

### 2.1. Structure Elucidation

**Figure 2:**
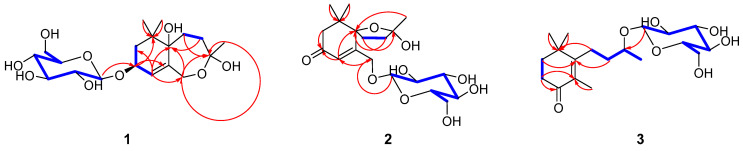
Key HMBC (H→C,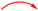) and ^1^H-^1^H COSY (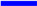) correlations of compounds **1**–**3**.

**Figure 3:**
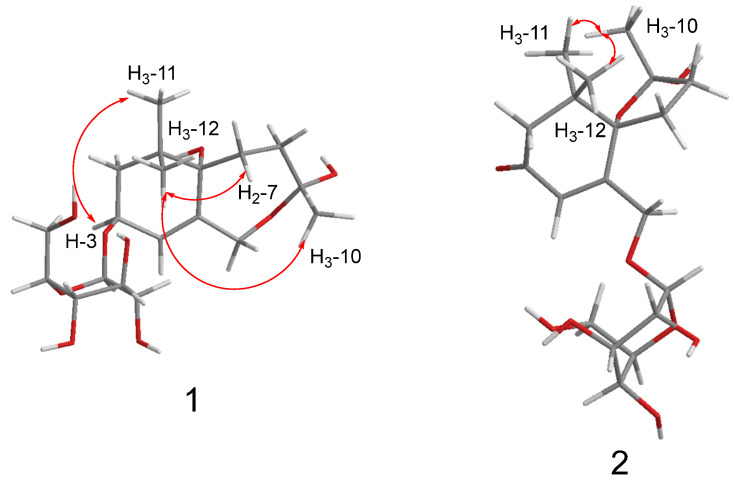
Key NOESY (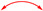) correlations of compounds **1** and **2**.

**Figure 4:**
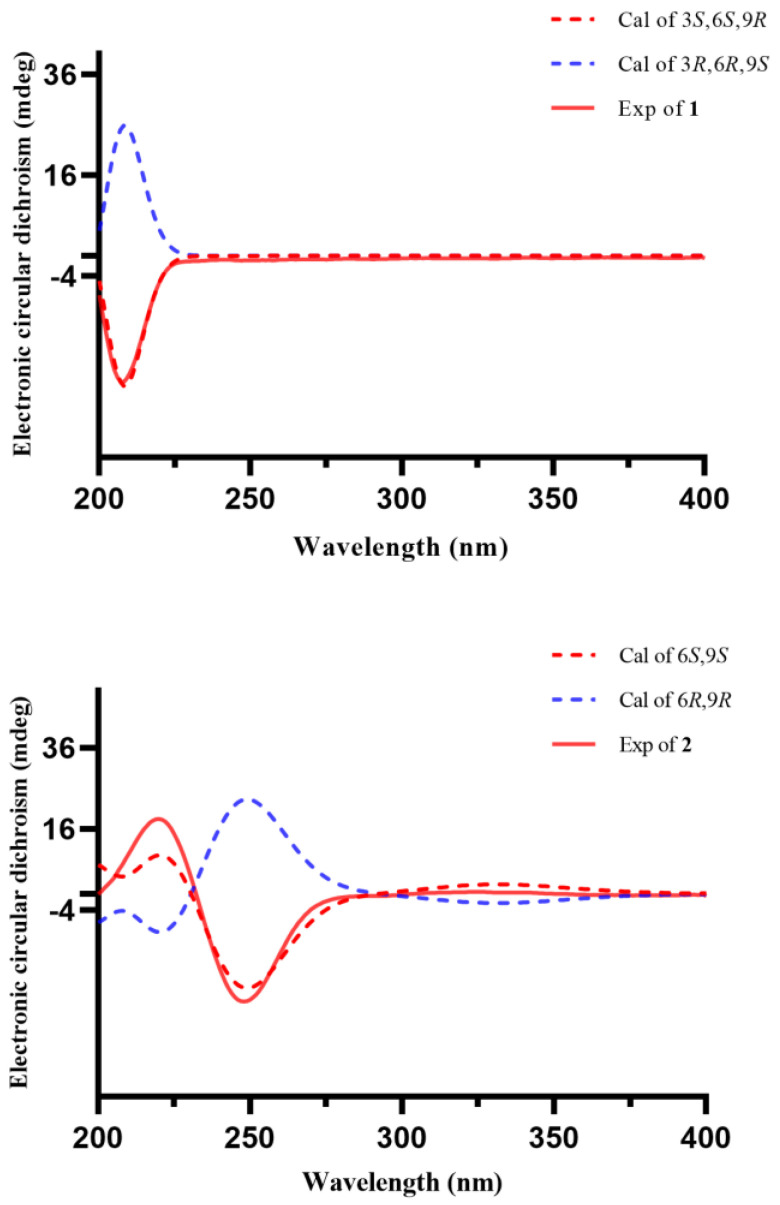
Experimental and calculated ECD curves of compounds **1** and **2**.

Compound **1** was obtained as colourless gum, and its molecular formula was established as C_19_H_32_O_9_ from its HRESIMS data (*m*/*z* 373.1937 [M + Na]^+^, calcd for C_19_H_32_O_9_Na, 427.1939). The ^1^H NMR spectrum of **1** (Table 1) displayed the signals of an olefinic proton at *δ*_H_ 5.77 (1H, br s, H-4), two oxygenated methylene protons at *δ*_H_ 4.48 (1H, d, *J* = 12.3 Hz, H-13a) and 4.02 (1H, d, *J* = 12.3 Hz, H-13b), an oxygenated methine proton at *δ*_H_ 4.36 (1H, dd, *J* = 6.4, 4.7 Hz, H-3), and three methyl groups at *δ*_H_ 1.39 (3H, s, H_3_-10), 0.99 (3H, s, H_3_-11), and 0.96 (3H, s, H_3_-12). The corresponding ^13^C NMR data (Table 1) showed 19 carbon signals, including one double bond pair at *δ*_C_ 124.8 (C-4) and 137.3 (C-5); four oxygenated carbons at *δ*_C_ 73.9 (C-3), 85.4 (C-6), 108.3 (C-9), and 66.7 (C-13); one quaternary carbon at *δ*_C_ 35.8 (C-1); three methylenes at *δ*_C_ 41.8 (C-2), 29.8 (C-7), and 35.7 (C-8); three methyls at *δ*_C_ 24.3 (C-10), 23.5 (C-11), and 24.1 (C-12); along with a *β*-glucose at *δ*_C_ 103.5 (C-1′), 75.1 (C-2′), 78.0 (C-3′), 71.6 (C-4′), 77.9 (C-5′), and 62.8 (C-6′). The sugar moiety was determined as D-glucose by LC-MS analyses after acid hydrolysis and derivatization. The ^1^H-^1^H COSY correlations (Figure 2) including H_2_-2/H-3/H-4 and H_2_-7/H_2_-8 led to the establishment of two coupled proton groups. The HMBC correlations (Figure 2) from H-4 to C-2/C-6/C-13, from H_2_-13 to C-4/C-6/C-9, from H_2_-7 to C-9, from H_2_-8 to C-6, and from H_3_-10 to C-8/C-13, combined with the degree of unsaturation, indicated that **1** was a bicyclic ionone glycoside and similar to 7,8-dihydro-3*β*,6*α*-dihydroxy-*α*-ionone 9-O-*β*-D-glucopyranoside [13], except the sugar was located at C-3 instead of C-9, and a hemiacetal structure was formed between C-9 and C-13. Those changes were verified via HMBC relationships (Figure 2) of H-1′/C-3, H_2_-13/C-9, and H_3_-10/C-13, respectively. Meanwhile, **1** was a novel ionone with a 6/7 bicyclic skeleton. In the NOESY spectrum (Figure 3), the correlations between H_3_-11 and H-3 indicated that OH-3 and H_3_-12 are located at the same side and regarded as *β*-oriented; the correlations between H_3_-12 and H_2_-7/H_3_-10 indicated that H_2_-7 and H_3_-10 are also *β*-oriented. So, the most probable configuration of **1** is either 3*S*, 6*S*, 9*R* or 3*R*, 6*R*, 9*S*. The absolute configurations at the stereogenic centres of **1** were determined to be 3*S*, 6*S*, 9*R* by comparison of the experimental ECD curve and calculated electronic circular dichroism (ECD) data at the B3LYP/6-311G(d,p) level in MeOH (Figure 4). Therefore, compound **1** was determined as shown in Figure 1, and named as capitsesqside A.

Compound **2** possessed the molecular formula C_19_H_30_O_9_, as determined by a quasi-molecular ion peak [M + Na]^+^ at *m*/*z* 425.1782 (calcd for C_19_H_30_O_9_Na, 425.1782) in its HRESIMS spectrum. The characteristic signals of an olefinic proton at *δ*_H_ 6.19 (1H, s, H-4); two oxygenated methylene protons at *δ*_H_ 4.73 (1H, dd, *J* = 18.4, 2.0 Hz, H-13a) and 4.54 (1H, dd, *J* = 18.4, 2.0 Hz, H-13b); and three methyl groups at *δ*_H_ 1.52 (3H, s, H_3_-10), 1.03 (3H, s, H_3_-12), and 1.01 (3H, s, H_3_-11) were presented in the ^1^H NMR spectrum. The ^13^C NMR and HSQC spectra (Table 1) showed that **2** contains a keto carbonyl group, one olefinic bond, three quaternary carbons, four methylenes, three methyls, and a glucose moiety. Furthermore, a *β*-glucose moiety was observed by the signals [*δ*_H_ 4.33 (1H, d, *J* = 7.7 Hz, H-1′), 3.26 (1H, m, H-2′), 3.36 (1H, m, H-3′), 3.36 (1H, m, H-4′), 3.26 (1H, m, H-5′), 3.86 (1H, dd, *J* = 12.0, 2.0 Hz, H-6′a), 3.68 (1H, dd, *J* = 12.0, 5.3 Hz, H-6′b)], and [*δ*_C_ 104.0 (C-1′), 75.0 (C-2′), 78.1 (C-3′), 71.6 (C-4′), 78.1 (C-5′), 62.7 (C-6′)]. The D-configuration of glucose was deduced based on LC-MS analyses after acid hydrolysis and derivatization. Combination with the ^1^H-^1^H COSY correlation (Figure 2) of H_2_-7/H_2_-8 as well as the HMBC correlations (Figure 2) from H_3_-11/H_3_-12 to C-1, H-4 to C-3/C-5/C-6, H_2_-7 to C-9, and H_2_-8/H_3_-10 to C-6 suggested that **2** was also a bicyclic ionone glycoside, and the aglycone of **2** was similar to (2*S*,5*S*)-2-hydroxy-2,6,10,10-tetramethyl-1-oxaspiro[4.5]dec-6-en-8-one [14]. The difference was that the CH_3_-13 was oxidised to CH_2_OH-13 in **2**, which can be proved by the chemical shifts of CH_2_OH-13 (*δ*_H_ 4.73/4.54, *δ*_C_ 67.9). The HMBC correlations of H-1′ with C-13 suggested that the sugar moiety linked at the C-13 (Figure 2). Relative configurations of the stereogenic centres in **2** were determined by the NOESY correlations (Figure 3) of H_3_-10 with H_3_-11/H_3_-12 [15]. Finally, the absolute configuration of compound **2** was defined as 6*S*,9*S* by comparison of the experimental and calculated ECD spectra (Figure 4). Consequently, the structure of **2** was finally determined as shown in Figure 1 and named as capitsesqside B.

Compound **3** was a colourless gum. The molecular formula of **3** was deduced by a quasi-molecular ion peak [M + Na]^+^ at *m*/*z* 395.2037 (calcd for C_19_H_32_O_7_Na, 395.2040) in the HRESIMS spectrum. The ^1^H NMR data (Table 1) showed the characteristic signals: an oxygenated methine proton at *δ*_H_ 3.97 (1H, m, H-9) and four methyl groups at *δ*_H_ 1.76 (3H, s, H_3_-13), 1.23 (3H, d, *J* = 6.2 Hz, H_3_-10), 1.20 (3H, s, H_3_-11), and 1.20 (3H, s, H_3_-12). The ^13^C NMR spectrum showed a total of 19 carbons, including four methyls, four methylenes, one methine, four quaternary carbons, and a glucose signal. The sugar moiety in **3** was also identified as D-glucose by LC-MS analyses after acid hydrolysis and derivatization. A comparison of the NMR data of **3** with those of phoebenoside A [16] indicated that they possess the same planar structures as hydroxymegastigman-5-en-4-one 9-O-*β*-D-glucopyranoside. The absolute configuration of C-9 in phoebenoside A was *S*. The chemical shifts of C-9 (*δ*_C_ 75.7), C-10 (*δ*_C_ 19.8), and C-1′ (*δ*_C_ 102.2) in **3** confirmed the 9*R* configuration by comparison with the corresponding data of (3*R*,9*S*)-megastigman-5-en-3,9-diol 9-O-*β*-D-glucopyranoside [C-9 (*δ*_C_ 77.9), C-10 (*δ*_C_ 21.8), and C-1′ (*δ*_C_ 103.9)] and (3*R*,9*R*)-megastigman-5-en-3,9-diol 9-O-*β*-D-glucopyranoside [C-9 (*δ*_C_ 76.1), C-10 (*δ*_C_ 19.8), and C-1′ (*δ*_C_ 102.2)] in the same solvent CD_3_OD [17]. Therefore, the structure of **3** was defined as (9*R*)-hydroxymegastigman-5-en-4-one 9-O-*β*-D-glucopyranoside and named as capitsesqside C.

The seven known compounds (**4**–**10**) isolated from *R. capitatum* were shown to be (5*R*,7*R*,10*S*)-isopterocarpolone *β*-D-glucopyranoside (**4**) [18], (6*R*,9*R*)-3-oxo-*α*-ionol *β*-D-glucopyranoside (**5**) [19], (6*R*,9*R*)-3-oxo-*α*-ionol glucosides (**6**) [19], (6*R*,9*S*)-3-oxo-*α*-ionol glucosides (**7**) [20], incarvilloside A (**8**) [21], incarvilloside B (**9**) [21], and 2*α*,3*α*,19*α*,24-tetrahydroxyurs-12-en-28-oic acid *β*-D-glucopyranosyl ester (**10**) [22].

### 2.2. Effect of Compounds ***1**–**10*** on the LPS-Induced Production of NO

**Figure 5:**
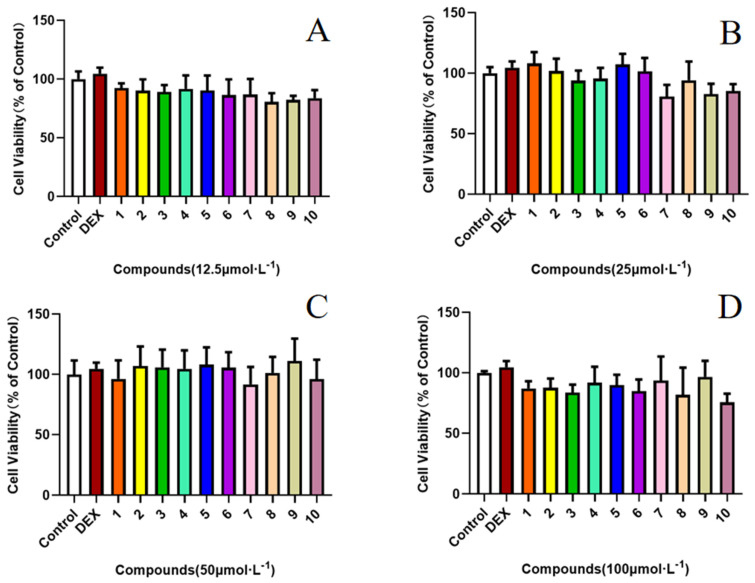
Effects of compounds **1**–**10** on cell viability ((**A**): 12.5 μM, (**B**): 25 μM, (**C**): 50 μM, and (**D**): 100 μM). The concentrations of these compounds ranged from 12.5 to 100 μM. Analysis of cell viability was by GraphPad Prism (8.0.2), and data are expressed as the mean ± SD. Three independent experiments were performed. Dexamethasone (DEX) was used as a positive control.

**Figure 6:**
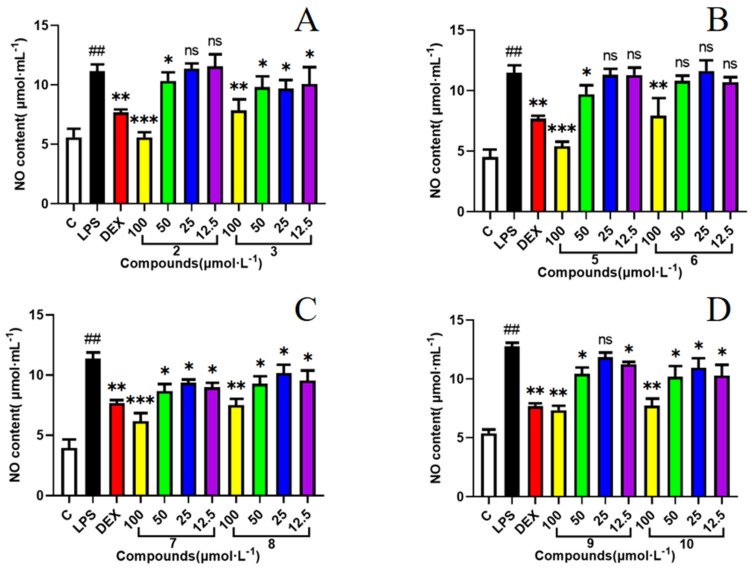
Effects of compounds **2**, **3** and **5**–**10** on LPS-induced production of NO in RAW 264.7 cells. The concentrations of these compounds ranged from 12.5 to 100 μM ((**A**): compounds **2** and **3**, (**B**): compounds **5** and **6**, (**C**): compounds **7** and **8**, and (**D**): compounds **9** and **10**). Analysis of cell viability was by GraphPad Prism, and data are expressed as the mean ± SD. Compare with control, ## *p* < 0.01; compare with LPS, ns > 0.05, * *p* < 0.05, ** *p* < 0.01, *** *p* < 0.001. Three independent experiments were performed. Dexamethasone (DEX, 10 μM) was used as a positive control.

**Figure 7:**
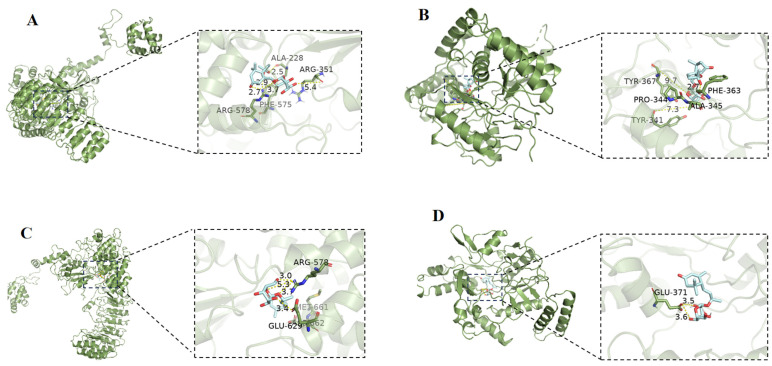
(**A**): The molecular interactions of compound **2** with NLRP3 by molecular docking simulation. (**B**): The molecular interactions of compound **2** with iNOS by molecular docking simulation. (**C**): The molecular interactions of compound **5** with NLRP3 by molecular docking simulation. (**D**): The molecular interactions of compound **5** with iNOS by molecular docking simulation.

The NOD-like receptor family pyrin domain containing 3 (NLRP3) protein is a member of the inflammatory vesicle protein family, and aberrant activation of this protein has been implicated in the pathogenesis of several inflammatory diseases. Inducible nitric oxide synthase (iNOS) and its product NO play an important role in the inflammatory response and oxidative stress, which is one of the major molecular mechanisms of inflammation. The MTT assay was used to examine the cytotoxicity of compounds **1**–**10** in RAW 264.7 cells. The results showed no significant cytotoxicity of any compounds at the testing concentrations (Figure 5). Compounds **1**–**10** were examined for inhibition of NO production in LPS (lipopolysaccharide)-induced RAW 264.7 cells by using the Griess method (Figure 6). In comparison with the positive control, the compounds **2** and **5** exhibited stronger NO inhibitory activity, and other compounds showed a slight inhibitory effect on NO production (Figure 6). The results of the molecular docking analysis indicated that compound **2** exhibited a high affinity for NLRP3 and iNOS, with binding energies of −6.52 and −6.64 kcal/mol, respectively (Figure 7). The carbonyl group at C-3 and hydroxy group at C-9 interact by hydrogen bonding in the NLRP3 pocket with amino acid residues PHE-575 and ALA-228, respectively. Moreover, the hydroxy group at C-9 also forms a hydrogen bonding interaction with PHE-363 in the iNOS pocket. However, compound **5** interacts more weakly than compound **2** with NLRP3 and iNOS.

## 3. Materials and Methods

### 3.1. General Experimental Procedures

UV and CD spectra were performed on an Applied Photophysics Chirascan spectrometer (Applied Photophysics Ltd., Surrey, UK). Optical rotations were determined using an Auton Paar MCP300 automatic polarimeter (Anton Paar GmbH, Graz, Austria). A Bruker AM-400 spectrometer with tetramethylsilane (TMS) as internal standard was used to acquire 1D and 2D NMR spectra. HRESIMS data were performed on a QTOF 6600 mass spectrometer (AB-SCIEX, Foster City, CA, USA). The TripleTOF 6600 UPLC/MS spectrometer (AB-SCIEX, Foster City, CA, USA) with a Kinetex C18 100Å column (2.6 μm, 2.1 × 50 mm) was used to analyse the test sample and standard sugar, coupled to an electrospray ionisation (ESI) interface. Sephadex LH-20 (GE Healthcare, Boston, MA, USA), MCI gel CHP20, and silica gel (100~200 mesh, 200~300 mesh, Qingdao Marine Chemical Co., Ltd., Qingdao, China) were employed for column chromatography (CC). Semipreparative HPLC was achieved on an Agilent 1100 series system using a 9.4 mm × 250 mm, 5 μm, YMC C18 column. Thin-layer chromatography (TLC) was performed on silica gel 60F254 and RP-18 F254S plates (Merck KGaA, Darmstadt, Germany). Reagents for the glucose discrimination (L-cysteine methyl ester hydrochloride and *o*-tolyl isothiocyanate) were purchased from Shanghai Macklin Biochemical Co., Ltd. (Shanghai, China), and D-glucuronic acid was obtained from Shanghai Yuanye Biotechnology Co., Ltd. (Shanghai, China). All other chemicals utilised in this study were of analytical grade.

### 3.2. Plant Material

The aerial parts of *R. capitatum* were collected in Mount Taibai, Shaanxi Province, in June 2019 (GPS coordinates: 107°25′–107°52′ E, 33°55′–33°35′ N) and identified by Dr. Zhen-Hai Wu, College of Life Sciences, Northwest A&F University. A voucher specimen (No. WUK 0480711) could be found in the Herbarium of the College of Life Sciences, Northwest A&F University.

### 3.3. Extraction and Isolation

The dried and crushed aerial parts of *R. capitatum* (8.7 kg) were dried and crushed and extracted four times with MeOH (30 L) for 3 h each time, resulting in a crude extract (1.2 kg). The crude extract was then dissolved in a triple amount of H_2_O water and partitioned sequentially with equal volumes of petroleum ether, EtOAc, and *n*-butanol to obtain three partitions. The *n*-butanol fraction (450.0 g) was separated into six fractions (Fr.A1~Fr.A6) using a MCI gel CHP 20P column and eluted with EtOH (0%, 20%, 40%, 60%, 80%, and 95%). Silica gel flash column chromatography (FCC) with dichloromethane/MeOH (40:1 to 1:1) was used to purify Fr.A4 (130.0 g), resulting in four fractions: Fr.A4-1~Fr.A4-4. Fr.A4-2 (5.0 g) was then divided into five subfractions (Fr.A4-2-1~Fr.A4-2-5) using silica gel FCC with dichloromethane/MeOH (40:1 to 2:1). The major fraction, Fr.A4-2-3 (1.4 g), was further purified by Sephadex LH-20 column eluting with MeOH, resulting in four subfractions: Fr.A4-2-3-1~Fr.A4-2-3-4. The Fr.A4-2-3-1 (322.0 mg) fraction was separated by semipreparative HPLC (MeOH/H_2_O, 49%:51%; flow rate: 2 mL/min) to obtain compound **1** (8.0 mg; *t*_R_ = 18.5 min), compound **2** (4.0 mg; *t*_R_ = 22.8 min), compound **3** (23.0 mg; *t*_R_ = 25.6 min), compound **4** (7.0 mg; *t*_R_ = 28.9 min), compound **5** (5.1 mg; *t*_R_ = 15.7 min), compound **6** (3.1 mg; *t*_R_ = 20.2 min), and compound **7** (3.2 mg; *t*_R_ = 21.2 min). Fr.A5 (60.0 g) was separated on a silica gel column using dichloromethane/MeOH (20:1 to 2:1) elution to yield six subfractions (Fr.A5-1~Fr.A5-6). Fr.A5-5 (1.7 g) was subjected to Sephadex LH-20 column elution with MeOH to obtain subfractions Fr.A5-5-1~Fr.A5-5-5. Fr.A5-5-3 (640.0 mg) was then purified by semipreparative HPLC (MeOH/H_2_O, 50%:50%; flow rate: 2 mL/min) to yield compounds **8** (136.0 mg; *t*_R_ = 27.2 min), **9** (12.0 mg; *t*_R_ = 31.4 min), and **10** (28.0 mg; *t*_R_ = 33.8 min).

Capitsesqside A (**1**): Colourless gum; [*α*${]}_{D}^{20}$ + 25.4 (*c* 0.1, MeOH); UV (MeOH) *λ*_max_ (log *ε*): 200.0 (2.30) nm; CD (MeOH) *λ*_max_ (Δ*ε*) 206 (– 24.86); ^1^H and ^13^C NMR data see Table 1; HRESIMS [M + Na]^+^ *m*/*z* 427.1937 (calcd for C_19_H_32_O_9_Na, 427.1939).

Capitsesqside B (**2**): Colourless gum; [*α*${]}_{D}^{20}$ + 19.1 (*c* 0.1, MeOH); UV (MeOH) *λ*_max_ (log *ε*): 238.0 (2.39); CD (MeOH) *λ*_max_ (Δ*ε*) 220 (+ 18.10), 247 (– 22.34); ^1^H and ^13^C NMR data see Table 1; HRESIMS [M + Na]^+^ *m*/*z* 425.1782 (calcd for C_19_H_30_O_9_Na, 425.1782).

Capitsesqside C (**3**): Colourless gum; [*α*${]}_{D}^{20}$ + 22.0 (*c* 0.1, MeOH); UV (MeOH) *λ*_max_ (log *ε*): 200.0 (2.65), 245.0 (2.64) nm; ^1^H and ^13^C NMR data see Table 1; HRESIMS [M + Na]^+^ *m*/*z* 395.2037 (calcd for C_19_H_32_O_7_Na, 395.2040).

### 3.4. Acid Hydrolysis of Compounds ***1**–**3***

Compounds **1**–**3** (1 mg) were dissolved in 2 N HCl (3 mL) and stirred in water at 90 °C for 2.5 h. The resulting acid aqueous solutions were then concentrated under reduced pressure. To each residue, 1 mL of water was added, and the resulting solutions were extracted with 3 × 1 mL of EtOAc. The residue was dissolved in pyridine (1 mL) containing L-cysteine methyl ester hydrochloride (1 mg, Macklin, Shanghai, China) and stirred at 60 °C for 1.5 h. To each mixture, *o*-tolyl isothiocyanate (20.0 μL, Macklin, Shanghai, China) was added, and the resulting mixture was stirred at 60 °C for an additional 1.5 h. Finally, the compounds were analysed directly by LC-MS [23].

### 3.5. ECD Calculations

Gaussian 16 software was used to perform ECD calculations. Conformation optimization was carried out in the gas phase using density functional theory (DFT) at the B3LYP/6-31G(d) level. Time-dependent density functional theory was also employed. (TDDFT) ECD calculations were carried out in MeOH (PCM) using the B3LYP/6-311G(d,p) level, and ECD spectra were obtained with SpecDis 1.7 [24].

### 3.6. Cell Culture

The murine macrophage RAW 264.7 cells were obtained from the Institute of Materia Medica, Chinese Academy of Medical Sciences and Peking Union Medical College. They were regularly maintained at 37 °C in DMEM (Cbico, New York, NY, USA) containing 10% FBS (ABW, Frickenhausen, German) and 100 U/mL penicillin (Solarbio, Beijing, China) in a humidified 5% CO_2_ atmosphere.

### 3.7. MTT Assay for Cytotoxicity

The MTT assay was used to determine cytotoxicity after pretreatment with compounds **1**–**10**. RAW 264.7 cells were plated at a density of 8 × 10^3^ cells/well in a 96-well plate and incubated for 24 h before sample treatment. The cells were then pretreated with various concentrations of compounds (12.5, 25, 50, and 100 μM) for 24 h. Next, 10 μL of 5 mg/mL MTT was added to each well, followed by an additional 4 h incubation. The cultured medium was removed, and the formazan crystals were dissolved in 150 μL of DMSO. The absorbance was measured at 490 nm using a Multidetection microplate reader (BioTek Instruments, Inc., Winooski, VT, USA).

### 3.8. Measurement of NO

The concentration of nitrite was measured to indicate NO production using the Griess reaction. Briefly, RAW 264.7 cells were seeded into 96-well tissue culture plates at a density of 2 × 10^4^ cells/mL and stimulated with 1 μg/mL of LPS in the presence or absence of compounds. After incubation at 37 °C for 24 h, 50 μL of cell-free supernatant was mixed with 100 μL of Griess reagent. The mixture was then reacted at 37 °C for 10 min while avoiding light. Absorbance was measured at 550 nm against a calibration curve with sodium nitrite standards.

### 3.9. Molecular Docking

Molecular docking studies were performed to predict the binding interaction of compounds **2** and **5** to NLRP3 (PDB ID: 7PZC) and iNOS (PDB ID: 3E7G) using the software Autodock 4.2 Vina along with AutoDock Tools (ADT 1.5.6) according to the previously described method [25].

## 4. Conclusions

In conclusion, three novel ionone glycosides, capitsesqsides A−C (**1**–**3**), together with seven known compounds were isolated from *R. capitatum*. Among them, compound **1** was a rare 6/7 bicyclic skeleton ionone. Compounds **2** and **5** showed potent anti-inflammatory activity in LPS-induced NO production in RAW 264.7 cells. Compared to compounds **2**, **3**, and **5**–**7**, compound **1** showed reduced activity, suggesting that an *α*,*β*-unsaturated ketone group may be an active unit, while compound **4** and three triterpenoids (**8**–**10**) showed weak NO inhibitory activity. Molecular docking results suggested that NLRP3 and iNOS may be potential target proteins for the anti-inflammatory activity of compound **2**.

## Figures and Tables

**Table 1:** ^13^C NMR (100 MHz) and ^1^H NMR (400 MHz) data of **1**–**3** in CD_3_OD.

No.	1		2		3	
	*δ*_C_	*δ*_H_	*δ*_C_	*δ*_H_	*δ*_C_	*δ*_H_
1	35.8		43.0		37.6	
2	41.8	1.75 (m)	50.7	2.58 (d, 17.4) 1.20 (d, 17.4)	38.4	1.82 (t, 7.2)
3	73.9	4.36 (dd, 6.4, 4.7)	200.7		35.1	2.45 (t, 7.2)
4	124.8	5.77 (br s)	122.6	6.19 (s)	201.5	
5	137.3		168.1		131.6	
6	85.4		91.4	1.50 (m)	168.7	
7	29.8	2.10 (dd, 12.8, 4.6) 1.75 (m)	31.7	2.44 (m) 2.10 (dd, 14.5, 5.9)	27.9	2.53 (m) 2.31 (m)
8	35.7	2.19 (m) 1.96 (dd, 12.8, 4.6)	40.0	2.10 (dd, 14.5, 5.9) 2.01 (dd, 14.5, 9.6)	37.2	1.67 (m)
9	108.3		111.1		75.7	3.97 (m)
10	24.3	1.39 (s)	21.4	1.52 (s)	19.8	1.23 (d, 6.2)
11	23.5	0.99 (s)	23.3	1.01 (s)	27.2	1.20 (s)
12	24.1	0.96 (s)	24.9	1.03 (s)	27.2	1.20 (s)
13	66.7	4.48 (d, 12.3) 4.02 (d, 12.3)	67.9	4.73 (dd, 18.4, 2.0) 4.54 (dd, 18.4, 2.0)	11.7	1.76 (s)
1′-Gly	103.5	4.40 (d, 7.8)	104.0	4.33 (d, 7.7)	102.2	4.35 (d, 7.8)
2′-Gly	75.1	3.17 (m)	75.0	3.26 (m)	75.2	3.17 (dd, 9.0, 7.8)
3′-Gly	78.0	3.35 (m)	78.1	3.36 (m)	77.9	3.37 (m)
4′-Gly	71.6	3.28 (m)	71.6	3.36 (m)	71.8	3.37 (m)
5′-Gly	77.9	3.28 (m)	78.1	3.26 (m)	78.2	3.28 (m)
6′-Gly	62.8	3.86 (dd, 12.0, 2.2) 3.67 (dd, 12.0, 5.4)	62.7	3.86 (dd, 12.0, 2.0) 3.68 (dd, 12.0, 5.3)	62.9	3.88 (dd, 12.0, 2.0) 3.66 (dd, 12.0, 5.0)

## Data Availability

The data presented in this study are available in the Supplementary Materials.

## Supplementary Materials

The following supporting information can be downloaded at: https://www.mdpi.com/article/10.3390/molecules29112462/s1, Figures S1–S6: The 1D and 2D NMR spectra of compound **1** in CD_3_OD, Figure S7: The HRESIMS spectrum of compound **1**, Figures S8 and S9: The UV and ECD spectra of compound **1**, Figure S10: The LC-MS spectrum of the glucose moiety of compound **1**, Figures S11–S16: The 1D and 2D NMR spectra of compound **2** in CD_3_OD, Figure S17: The HRESIMS spectrum of compound **2**, Figures S18 and S19: The UV and ECD spectra of compound **2**, Figure S20: The LC-MS spectrum of the glucose moiety of compound **2**, Figures S21–S25: The 1D and 2D NMR spectra of compound **3** in CD_3_OD, Figure S26: The HRESIMS spectrum of compound **3**, Figure S27: The UV spectrum of compound **3**, Figure S28: The LC-MS spectrum of the glucose moiety of compound **3**, Figure S29: The LC-MS spectrum of the D-glucose.
