# Supplementary material for: Three New Ionone Glycosides from Rhododendron capitatum Maxim
Source: Molecules. 2024 May 23;29(11):2462. doi: 10.3390/molecules29112462 (PMC11173609; doi:10.3390/molecules29112462)
Supplement: Supplementary file 1 [file molecules-29-02462-s001.zip › molecules-2997890-supplementary.pdf]

Supplementary Materials

Three new ionone glycosides from *Rhododendron capitatum* Maxim

Jun-Ren Yang [1,2,†], Yue-Tong Zhu [1,†], Yi-Qin Zeng [1], Hong-Quan Li [1], Chun-Huan Li [1,*] and Jin-Ming Gao [1,*]

[1] Shaanxi Key Laboratory of Natural Products & Chemical Biology, College of Chemistry & Pharmacy, Northwest A&F University, Yangling 712100, Shaanxi, People's Republic of China
[2] Shaanxi Jiahe Phytochemistry Company, Xi'an 710077, China
* Correspondence: jinminggao@nwsuaf.edu.cn; chunhuanli@nwsuaf.edu.cn
† These authors have contributed equally to this work.

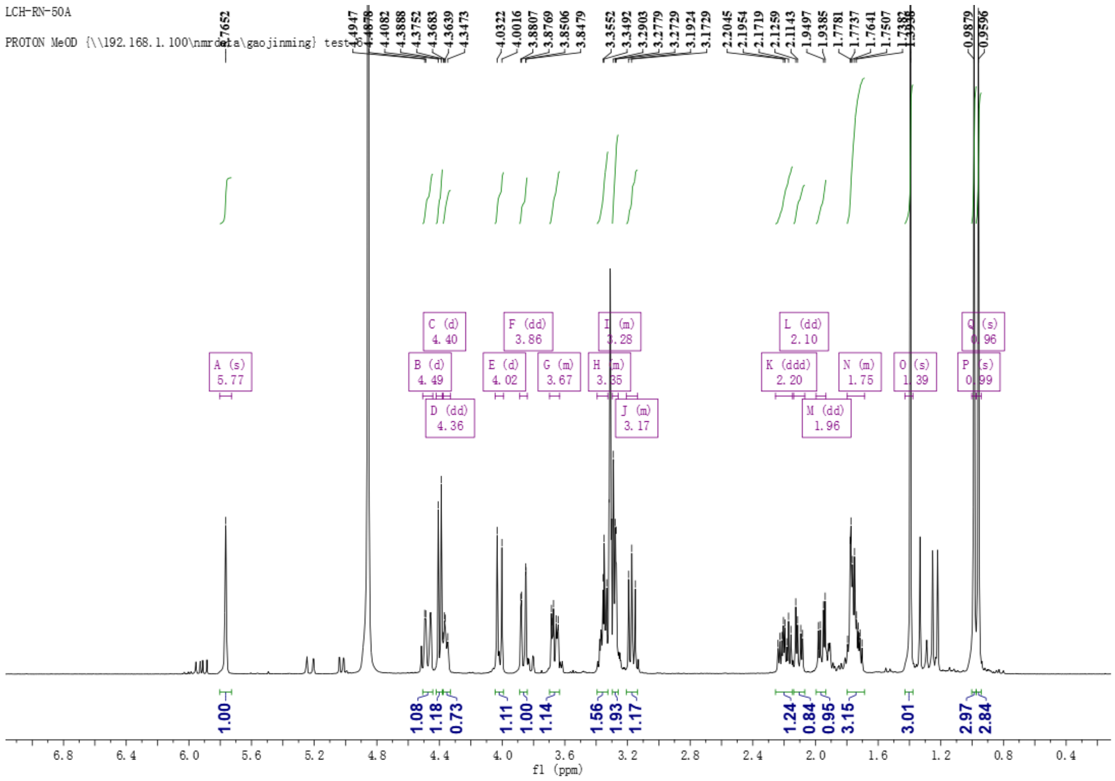

Figure S1. ^{1}H NMR (400 MHz) spectrum of compound **1** in methanol-d_4

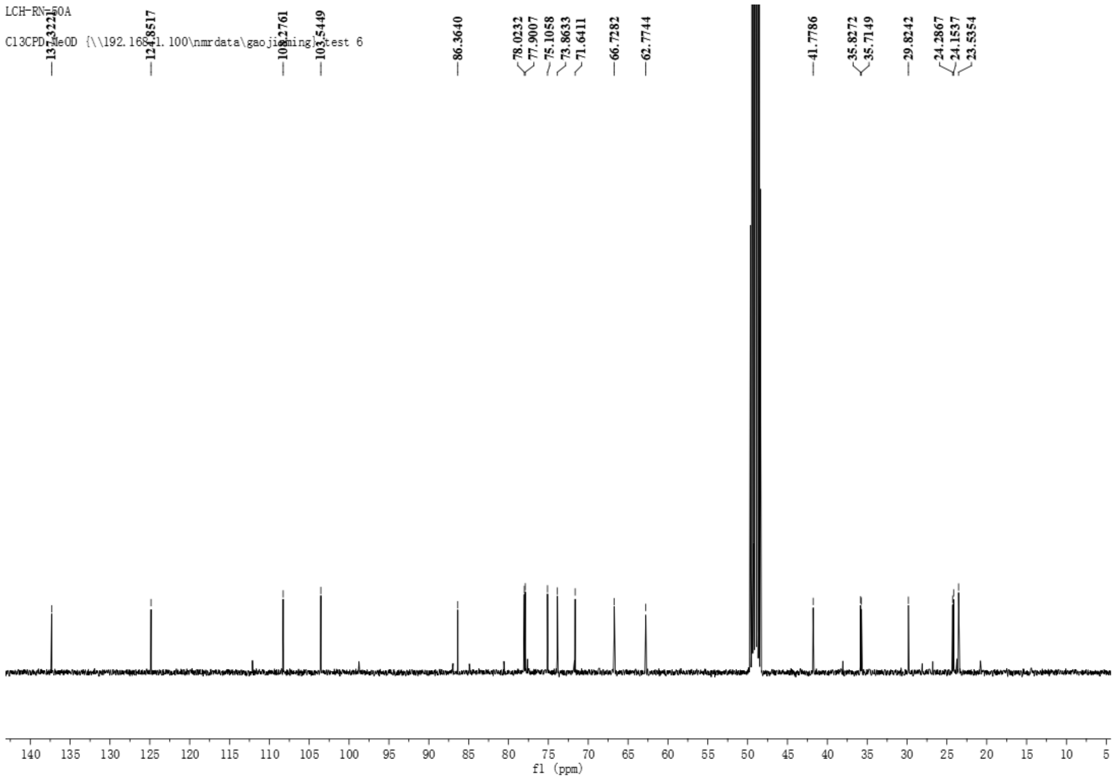

Figure S2. ^{13}C NMR (100 MHz) spectrum of compound **1** in methanol-d_4

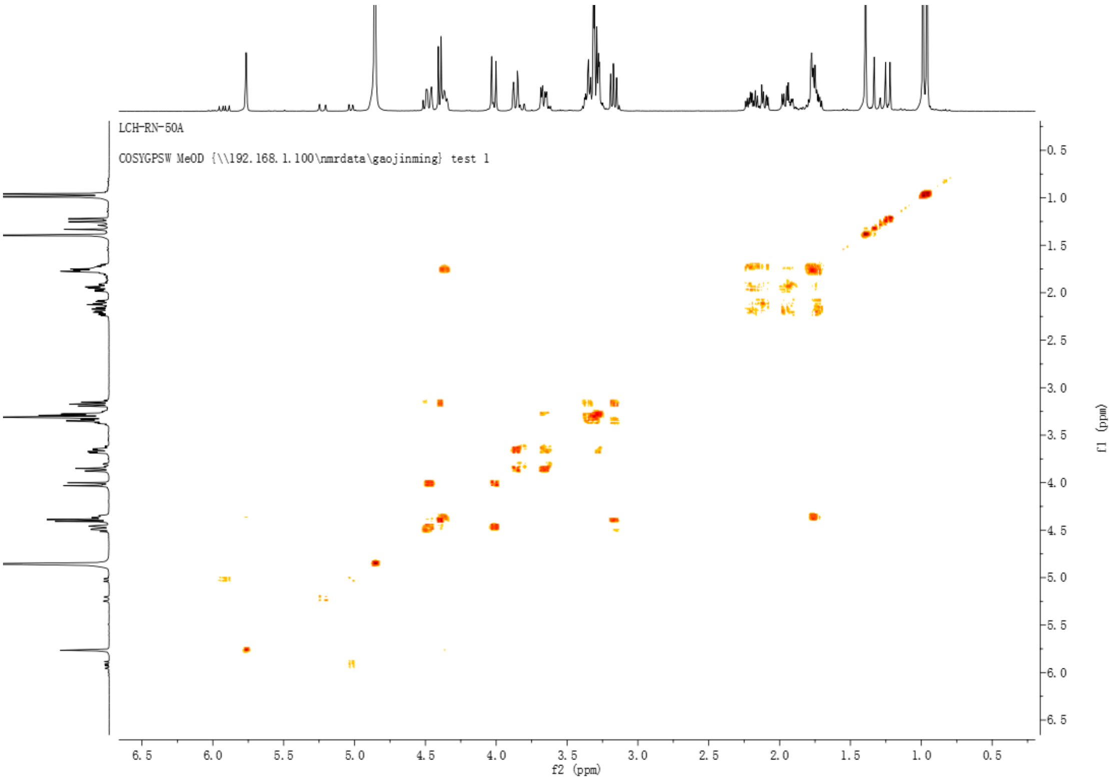

Figure S3. ^{1}H-^{1}H COSY (400 MHz) spectrum of compound **1** in methanol-d_4

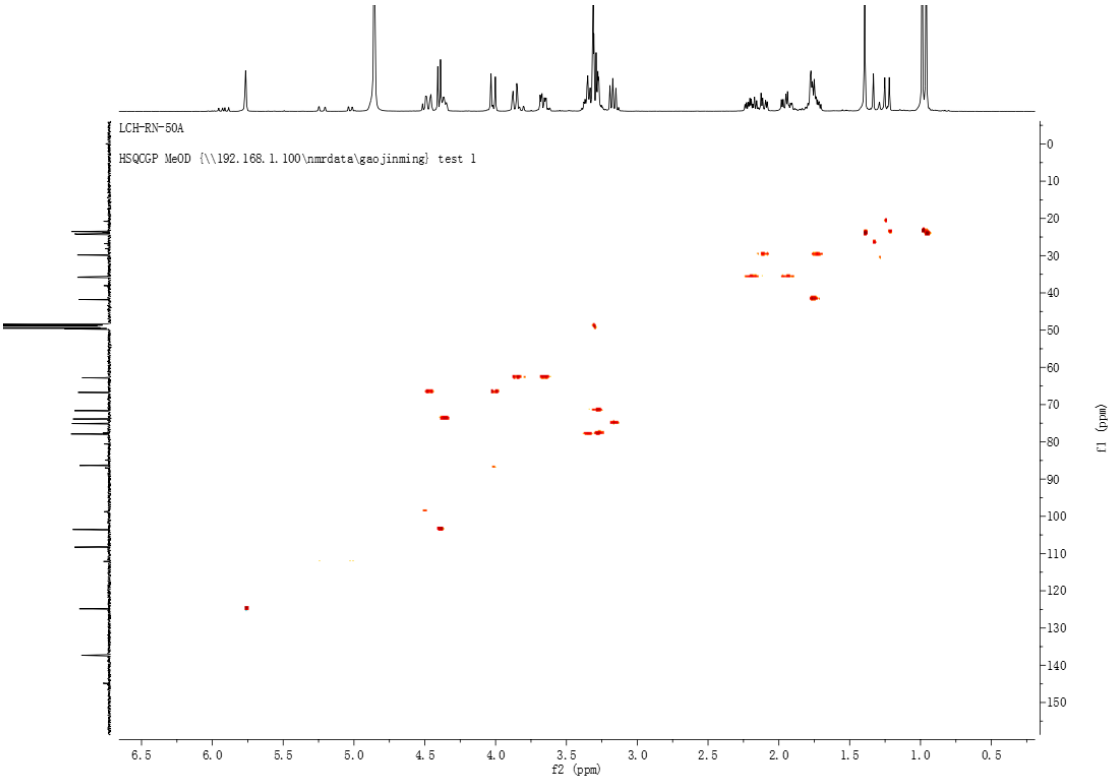

Figure S4. HSQC (400 MHz) spectrum of compound **1** in methanol-d_4

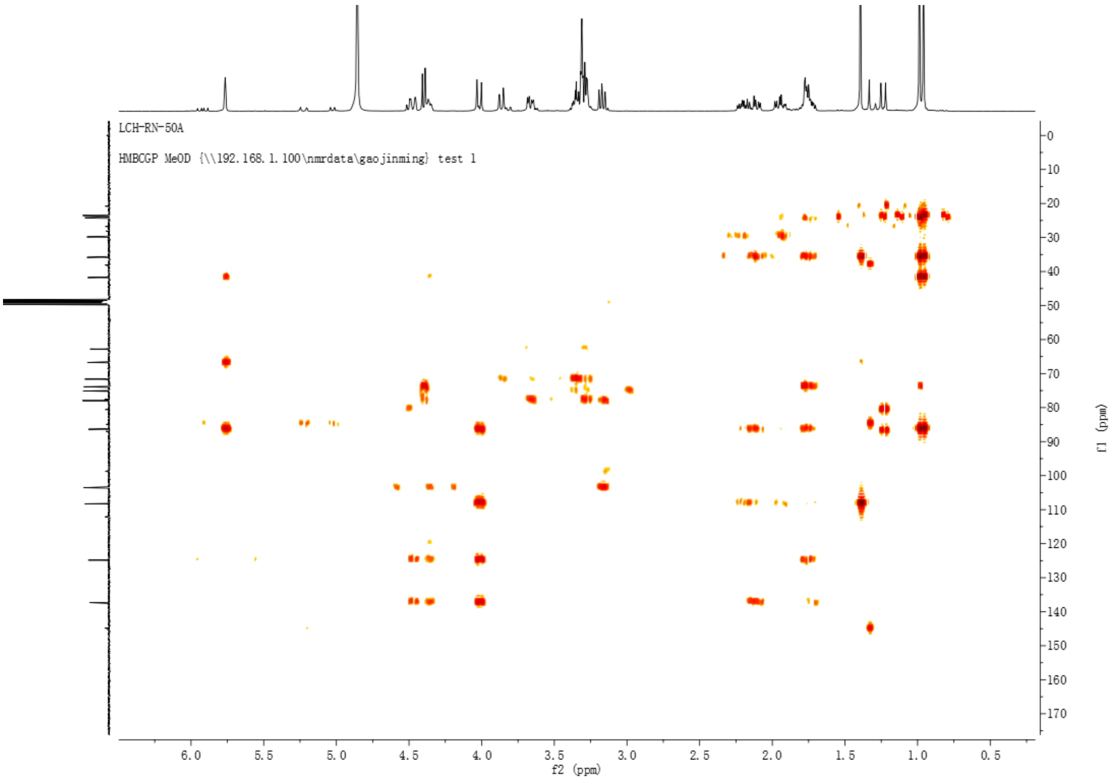

Figure S5. HMBC (400 MHz) spectrum of compound **1** in methanol-d_4

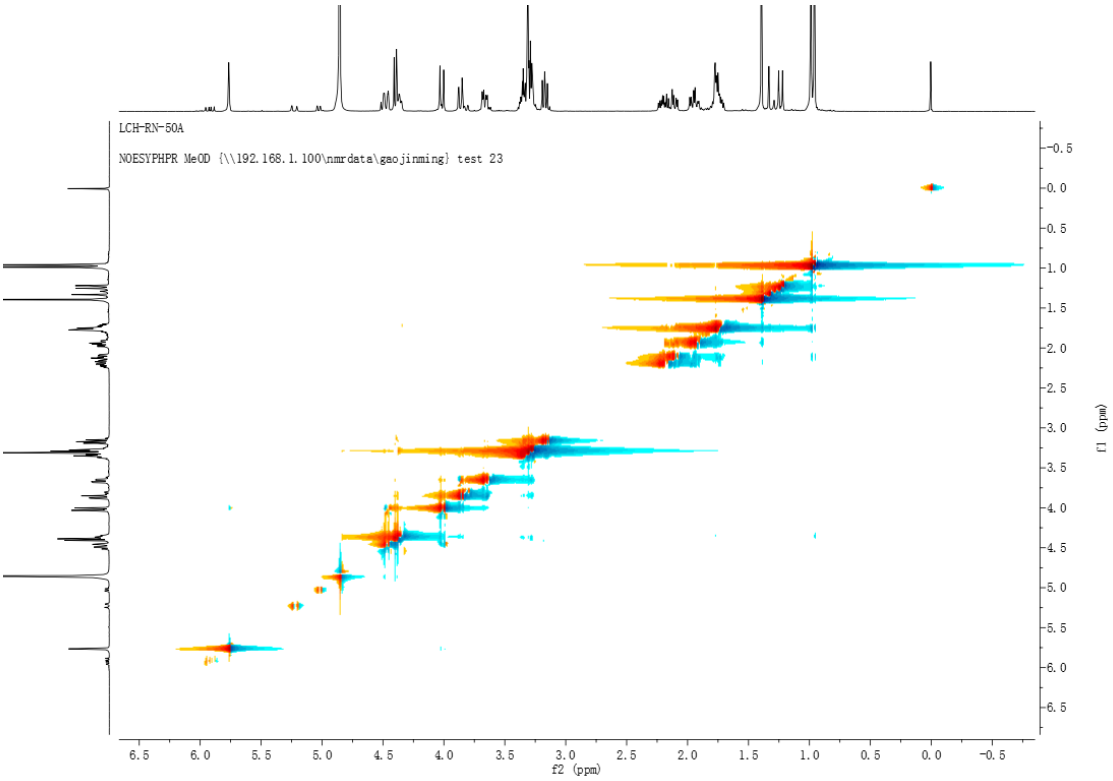

Figure S6. NOESY (400 MHz) spectrum of compound **1** in methanol-d_4

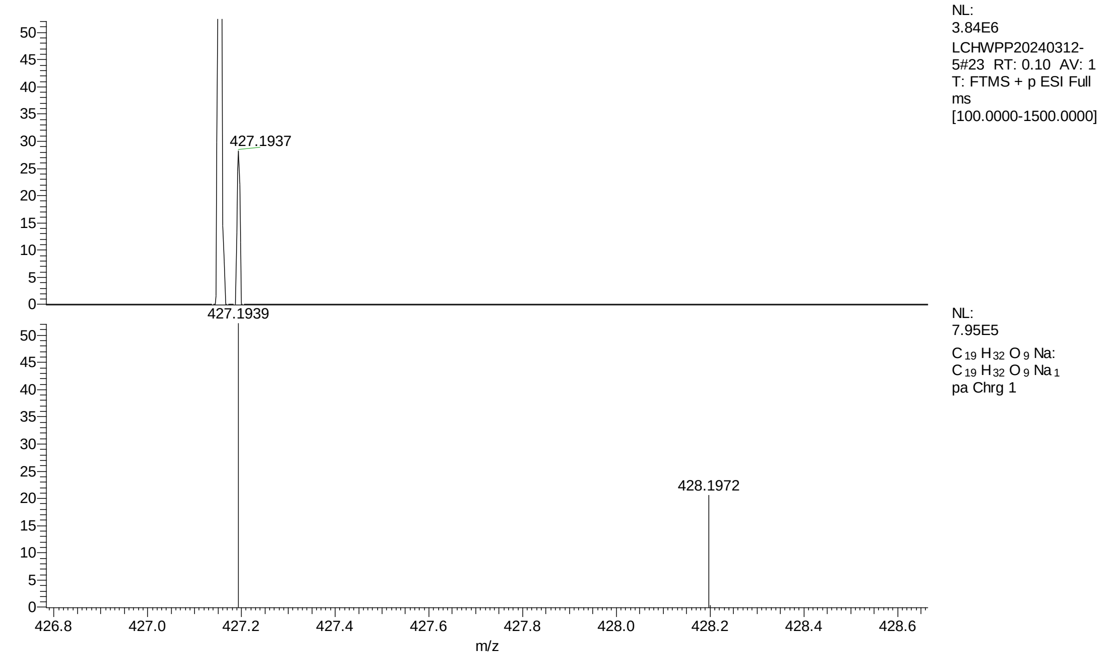

Figure S7. HRESIMS sof compound **1**

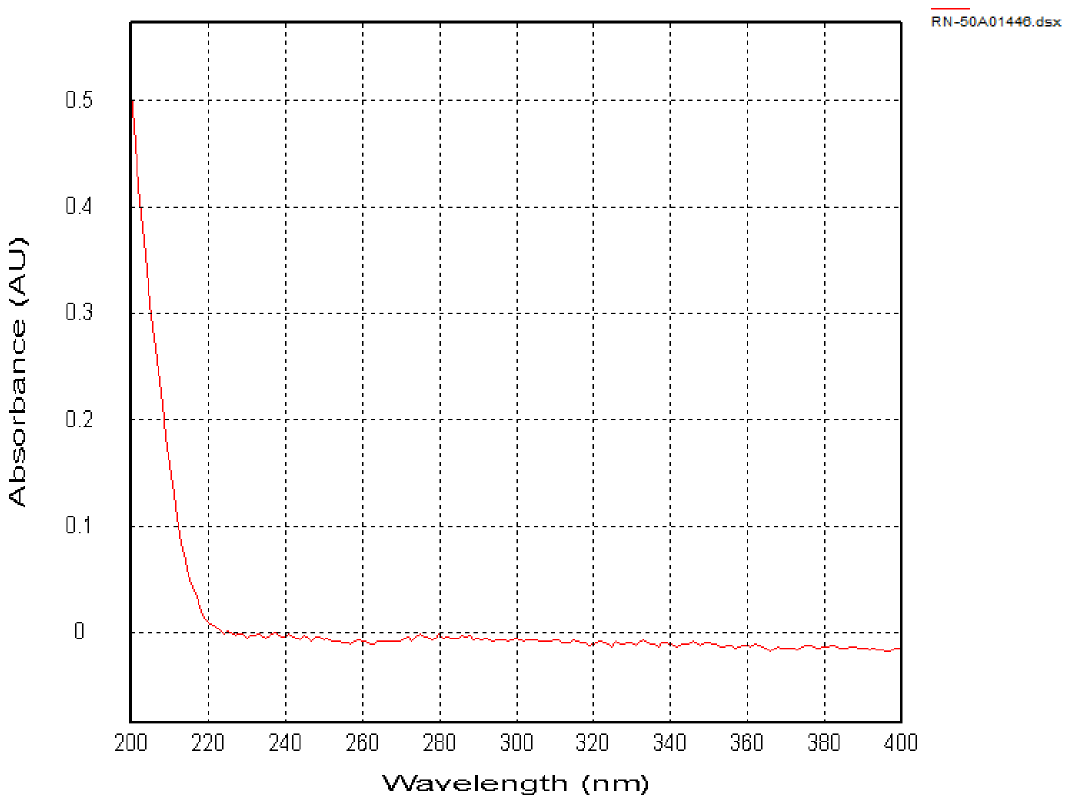

Figure S8. UV spectrum of compound **1** (MeOH)

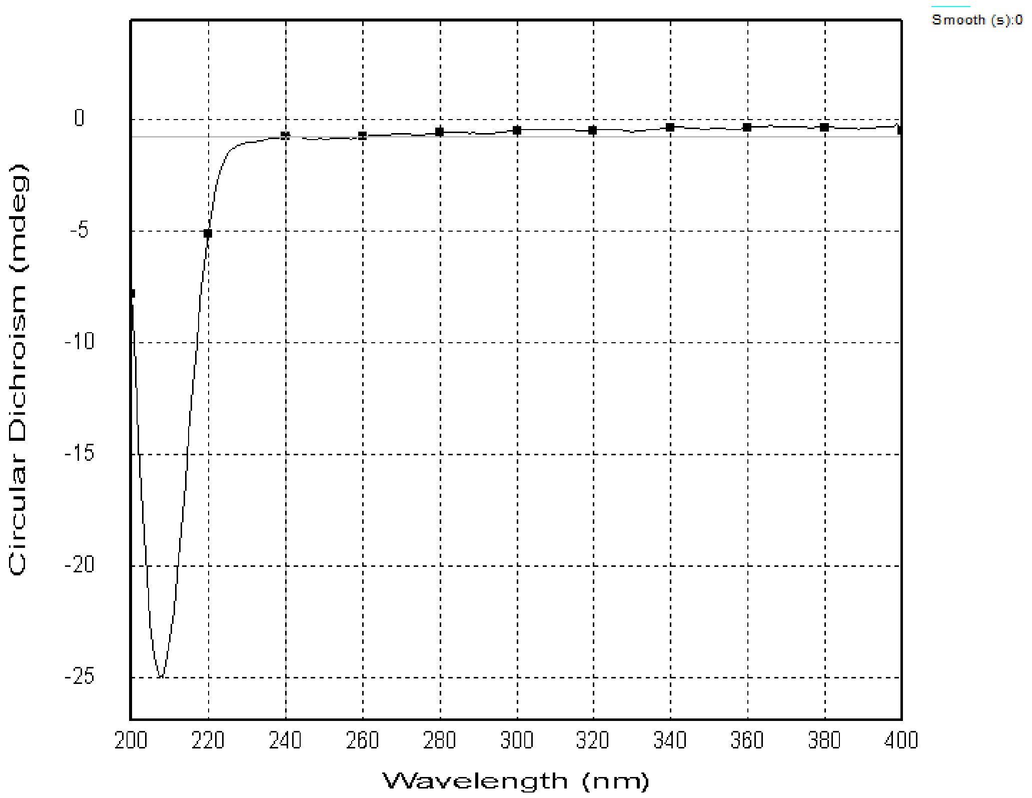

Figure S9. CD spectrum of compound **1** (MeOH)

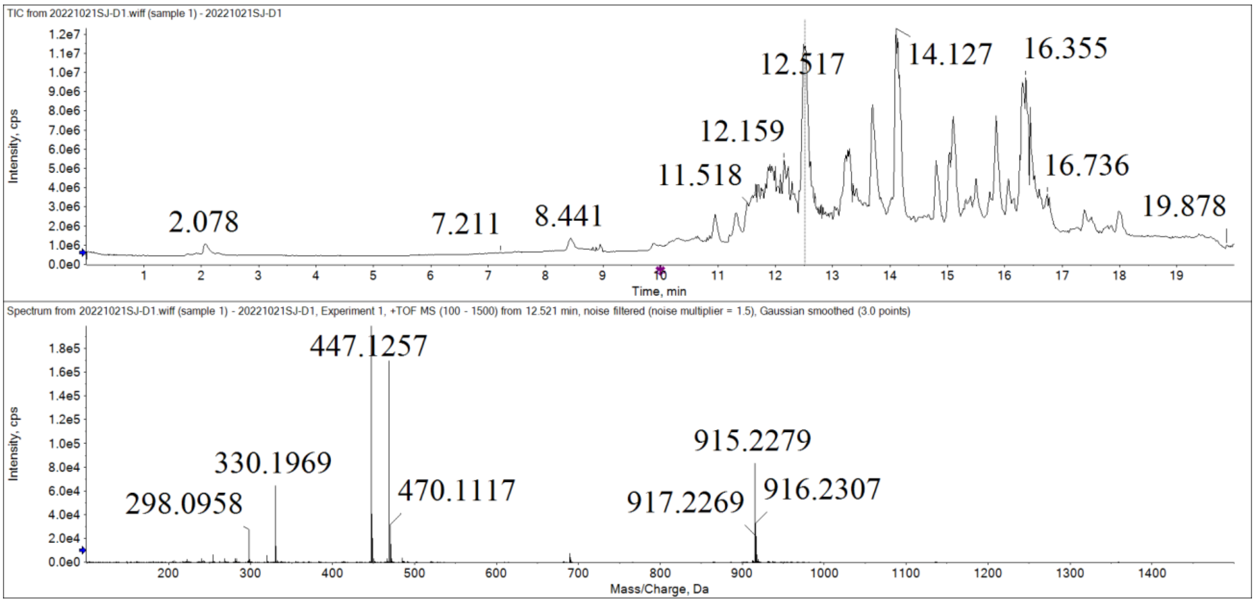

Figure S10. LC-MS spectrum of the glucose moiety of compound **1**

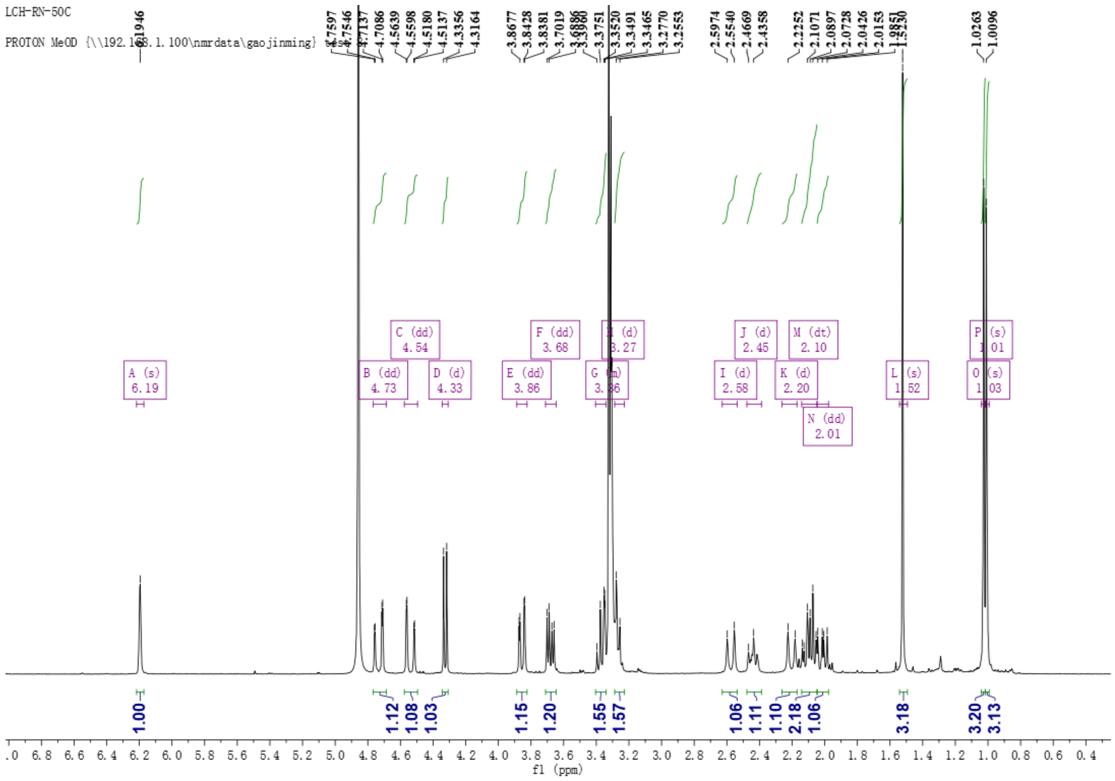

Figure S11. ^{1}H NMR (400 MHz) spectrum of compound **2** in methanol-d_4

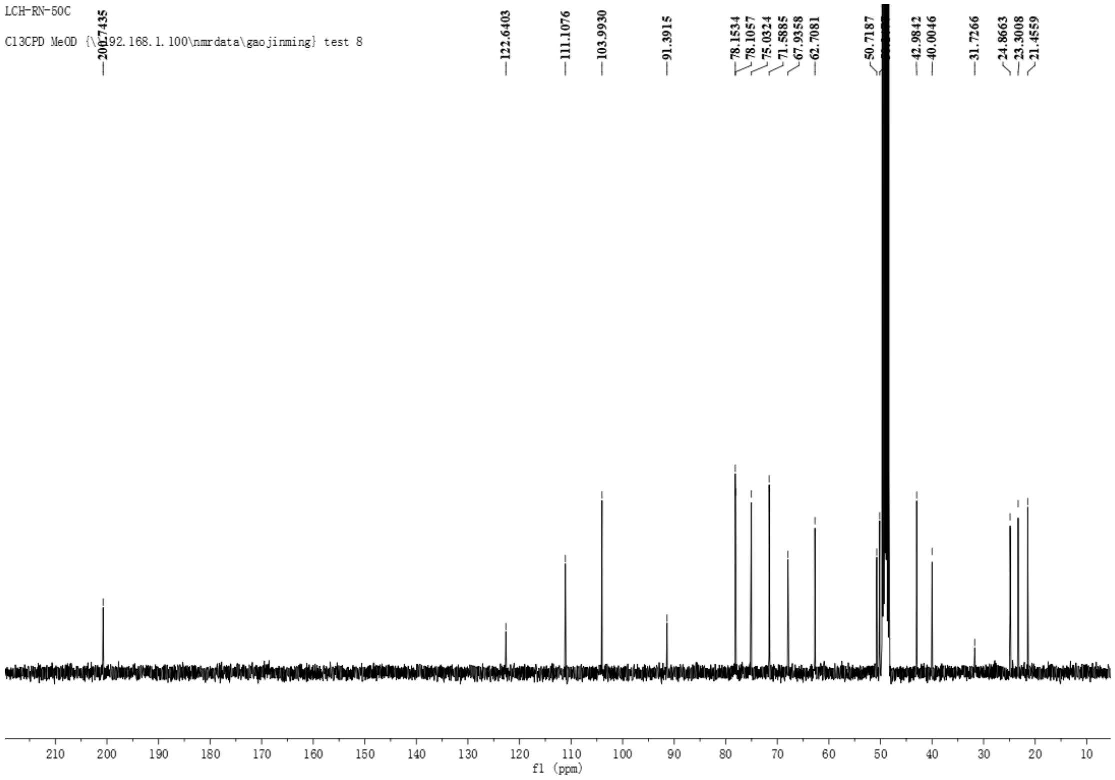

Figure S12. ^{13}C NMR (100 MHz) spectrum of compound **2** in methanol-d_4

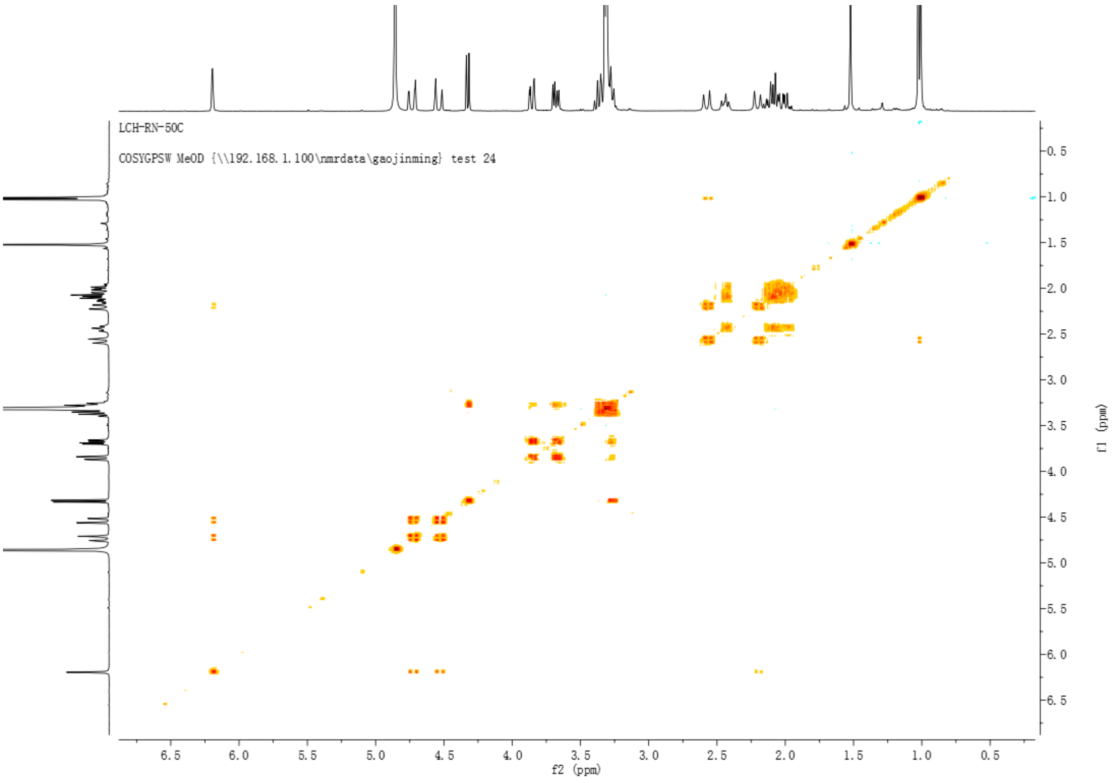

Figure S13. ^{1}H-^{1}H COSY (400 MHz) spectrum of compound **2** in methanol-d_4

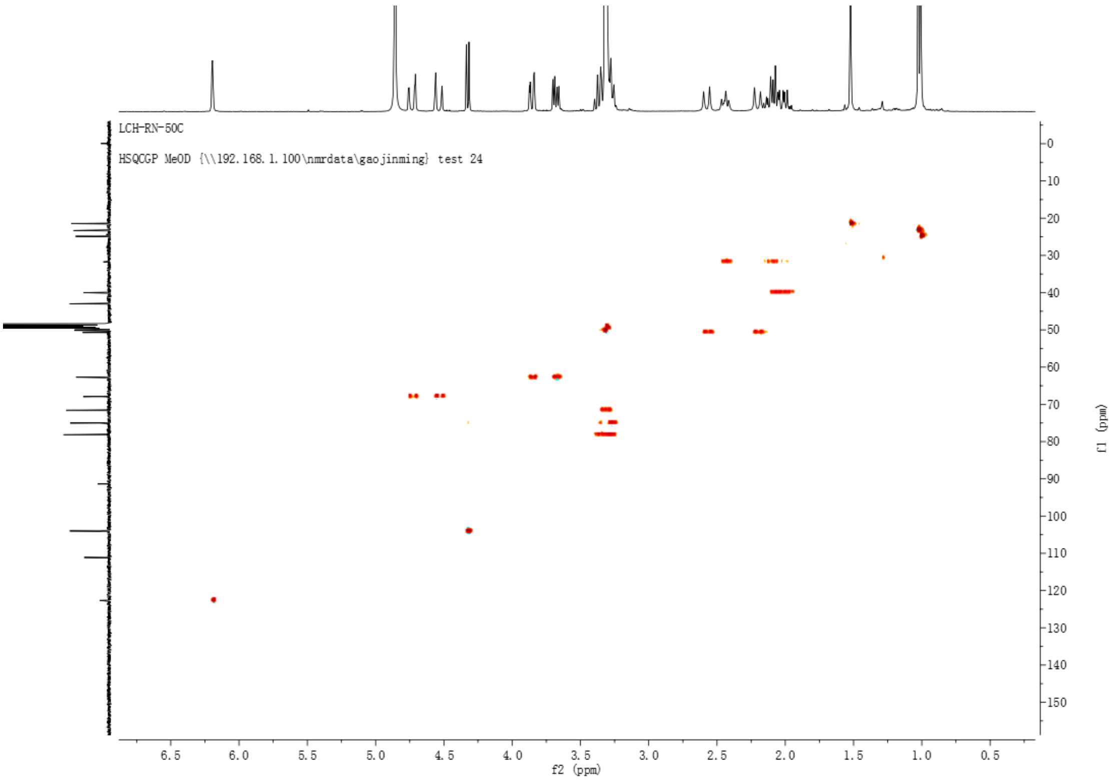

Figure S14. HSQC (400 MHz) spectrum of compound **2** in methanol-d_4

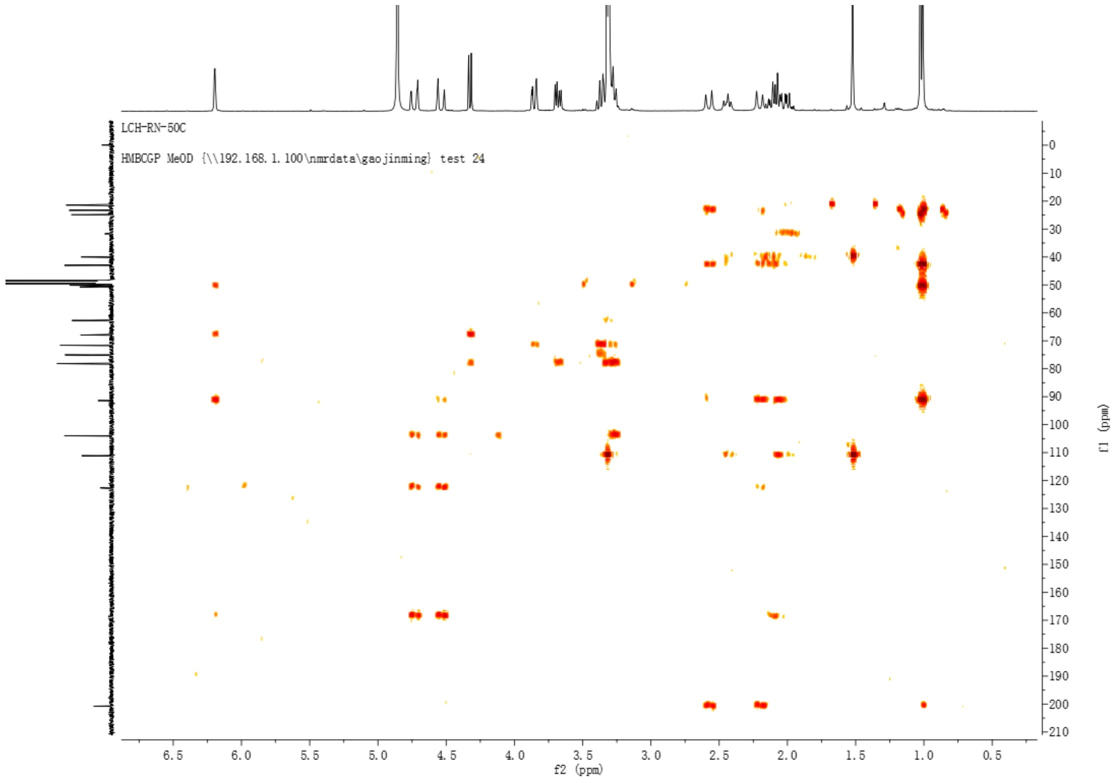

Figure S15. HMBC (400 MHz) spectrum of compound **2** in methanol-d_4

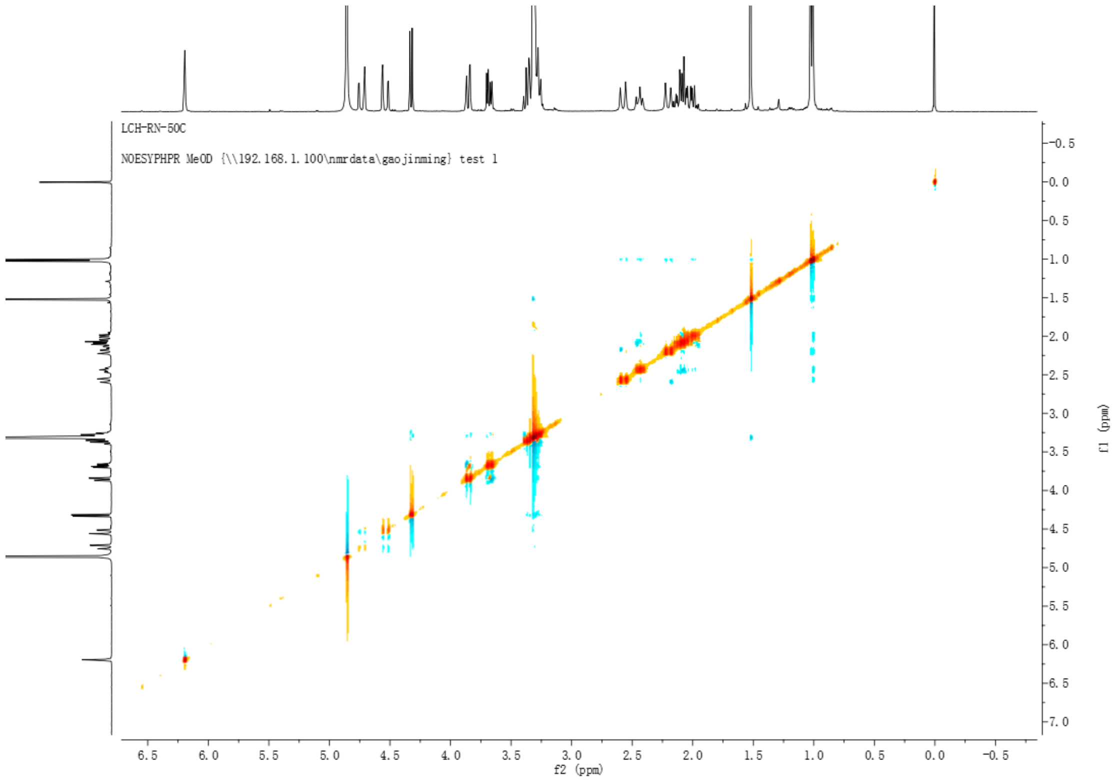

Figure S16. NOESY (400 MHz) spectrum of compound **2** in methanol-d_4

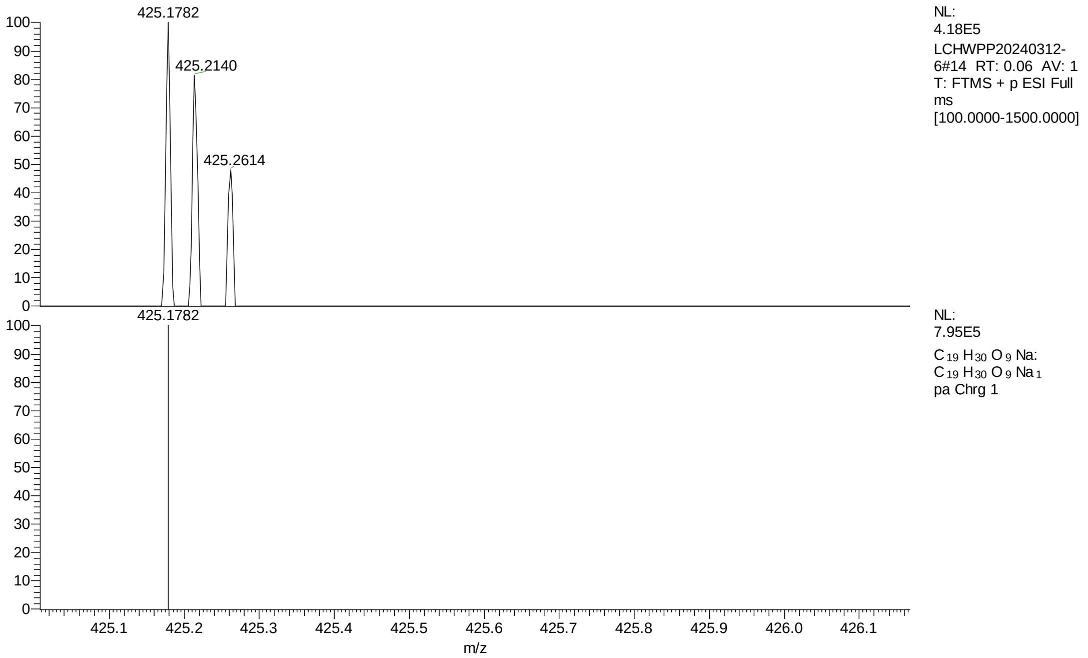

Figure S17. HRESIMS sof compound **2**

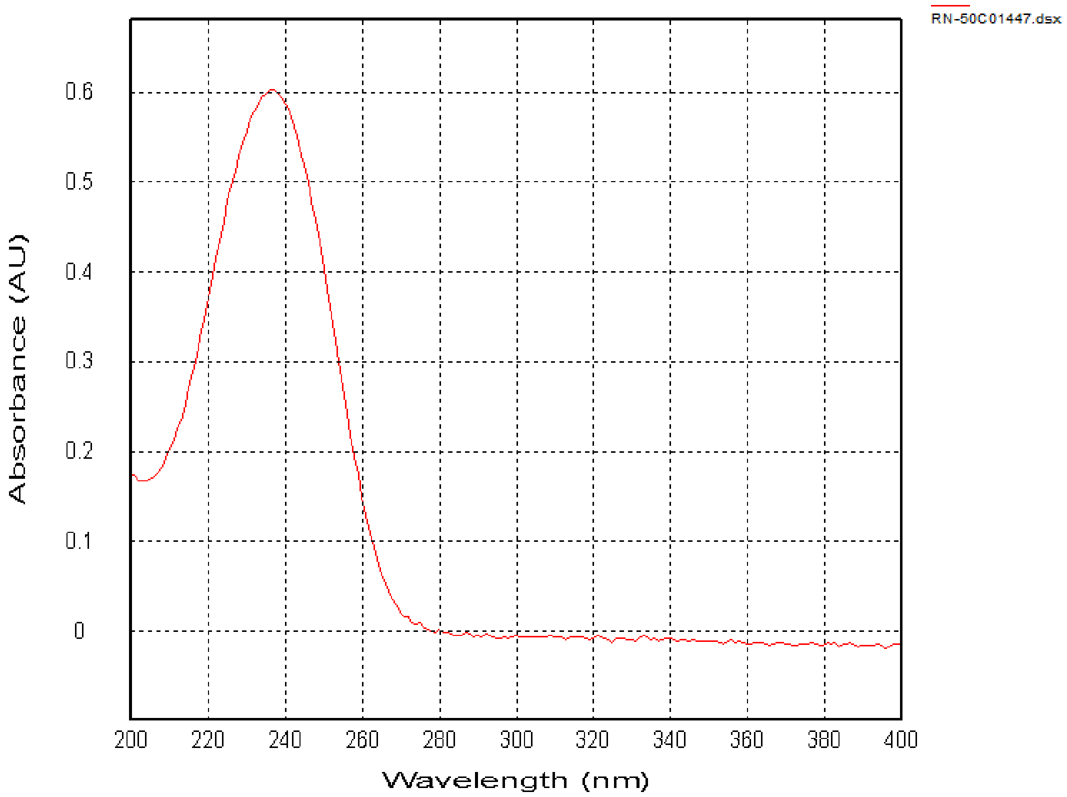

Figure S18. UV spectrum of compound **2** (MeOH)

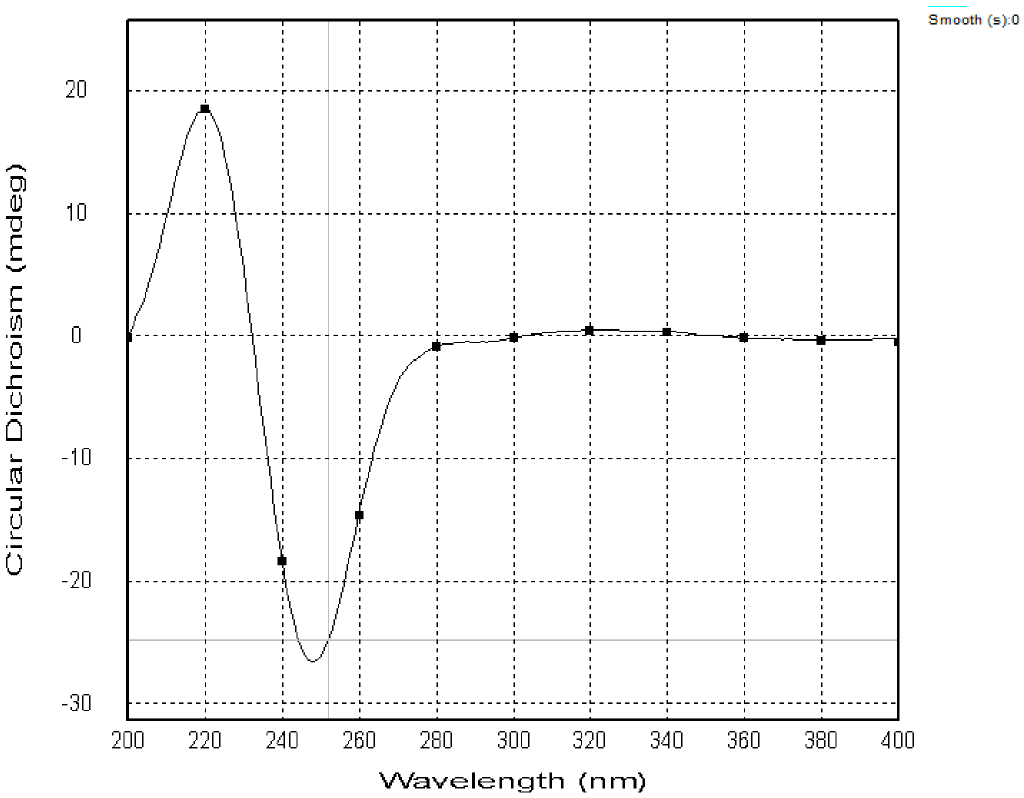

Figure S19. CD spectrum of compound **2** (MeOH)

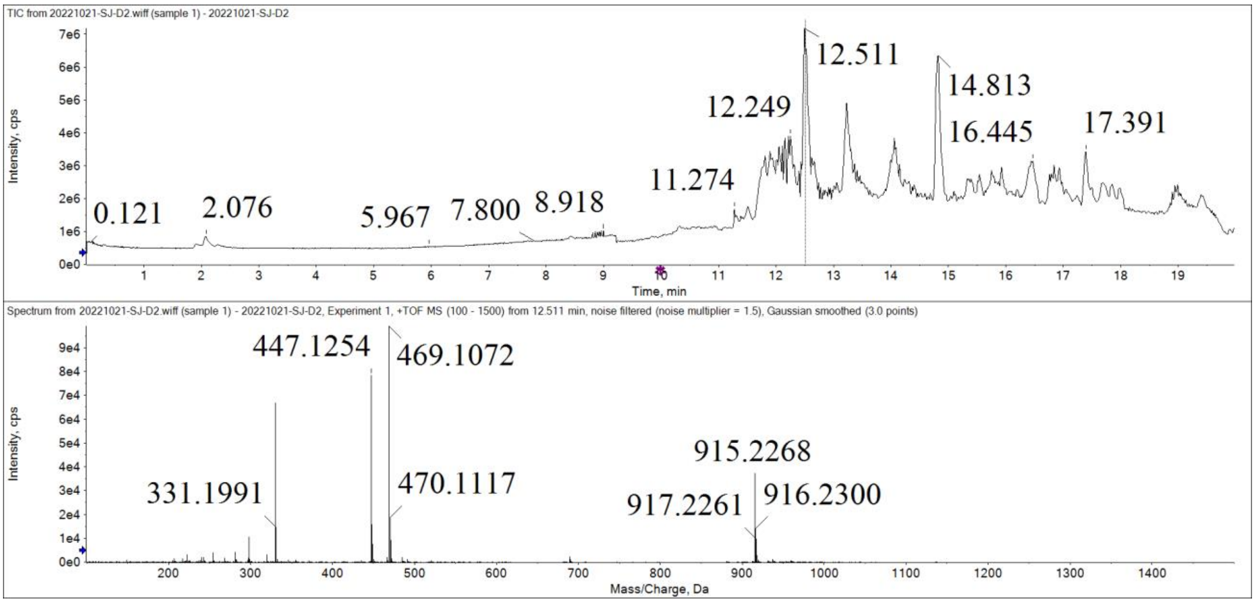

Figure S20. LC-MS spectrum of the glucose moiety of compound **2**

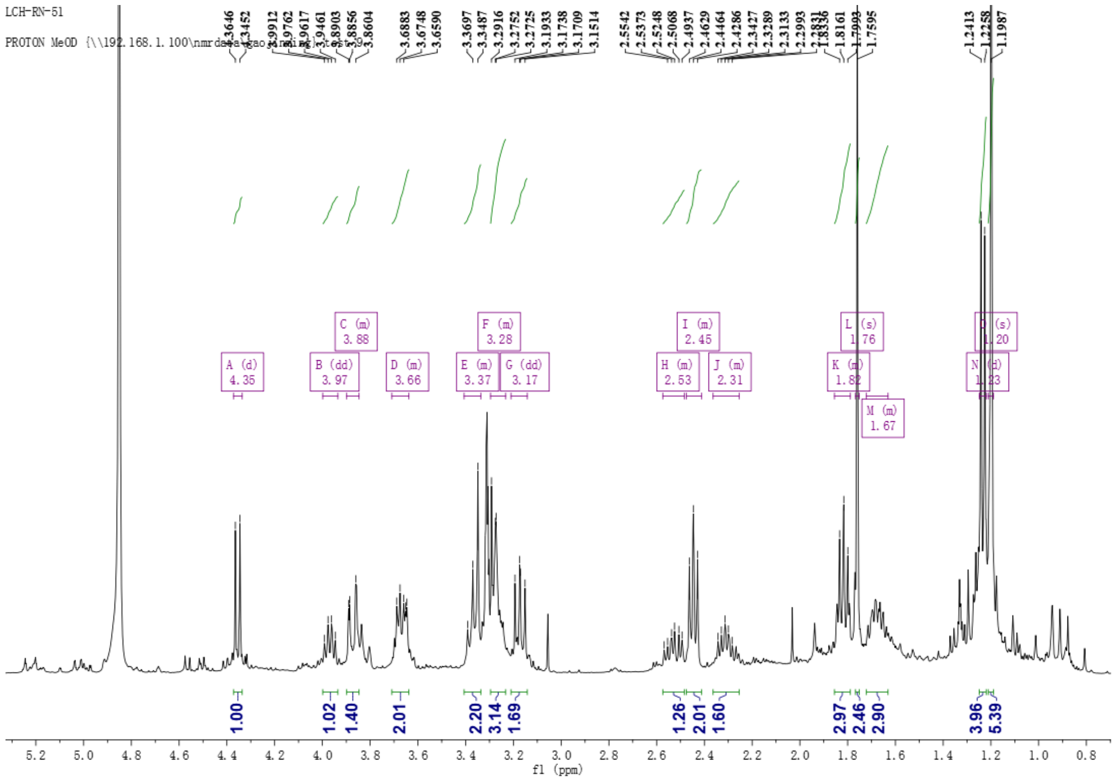

Figure S21. ^{1}H NMR (400 MHz) spectrum of compound **3** in methanol-d_4

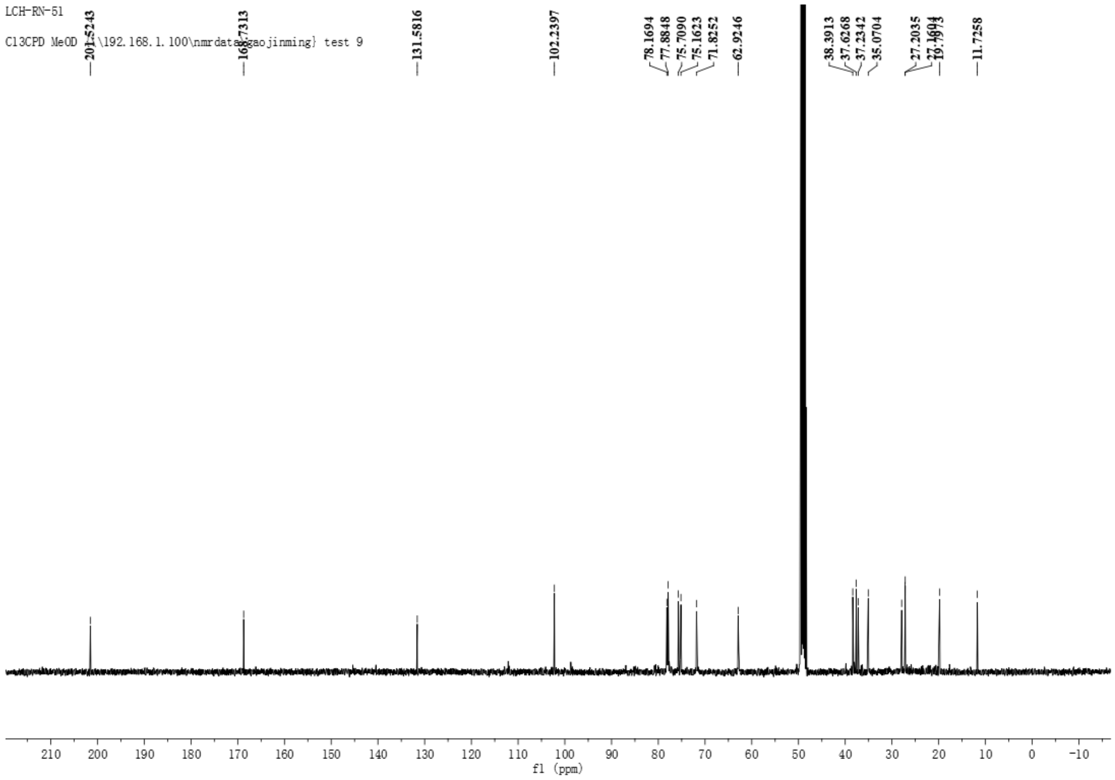

Figure S22. ^{13}C NMR (100 MHz) spectrum of compound **3** in methanol-d_4

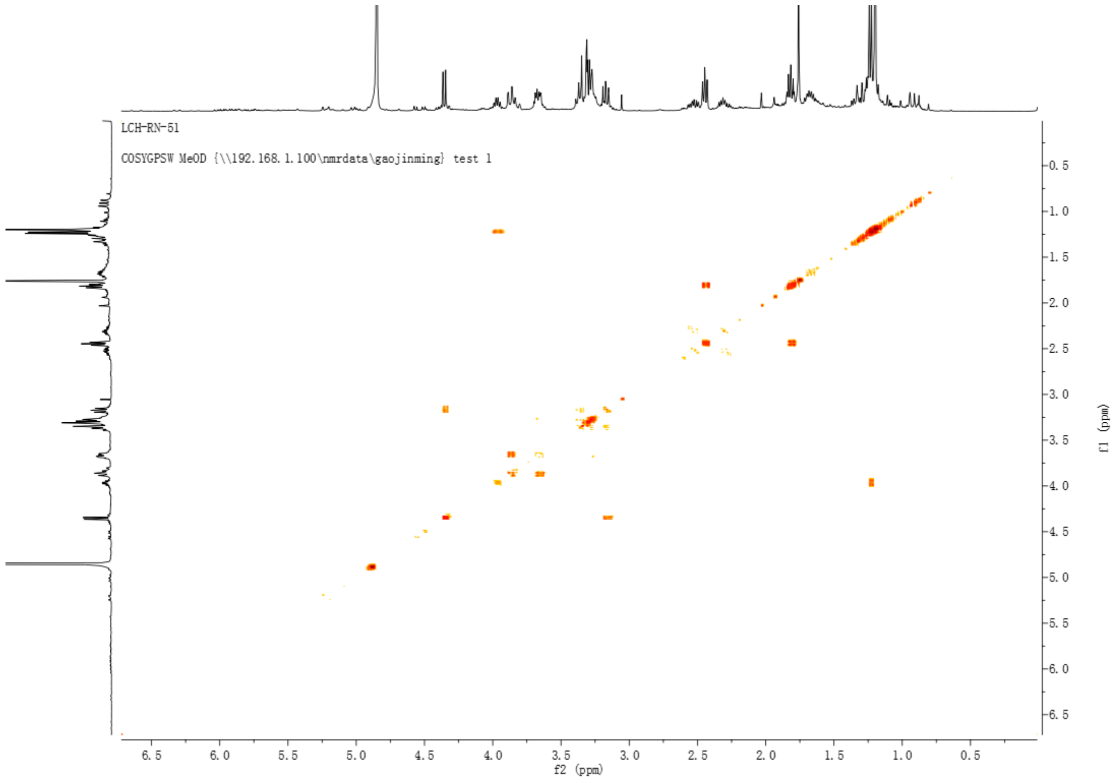

Figure S23. ${}^{1}H$-${}^{1}H$ COSY (400 MHz) spectrum of compound **3** in methanol-d_4

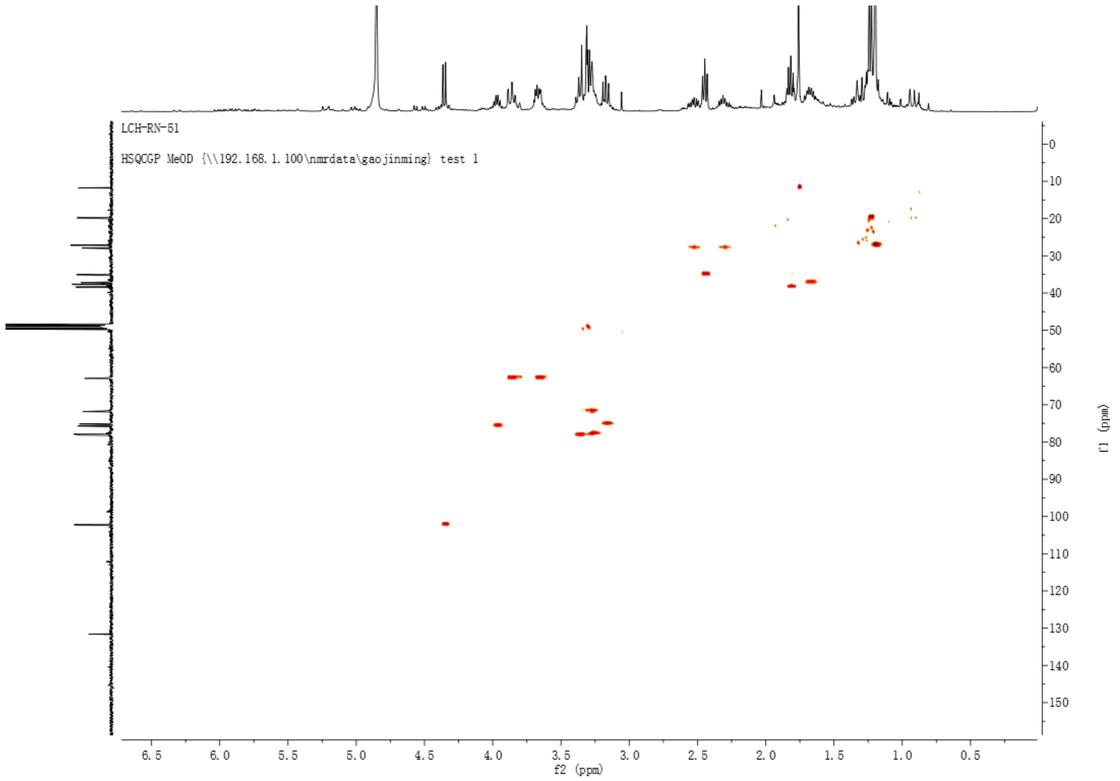

Figure S24. HSQC (400 MHz) spectrum of compound **3** in methanol-d_4

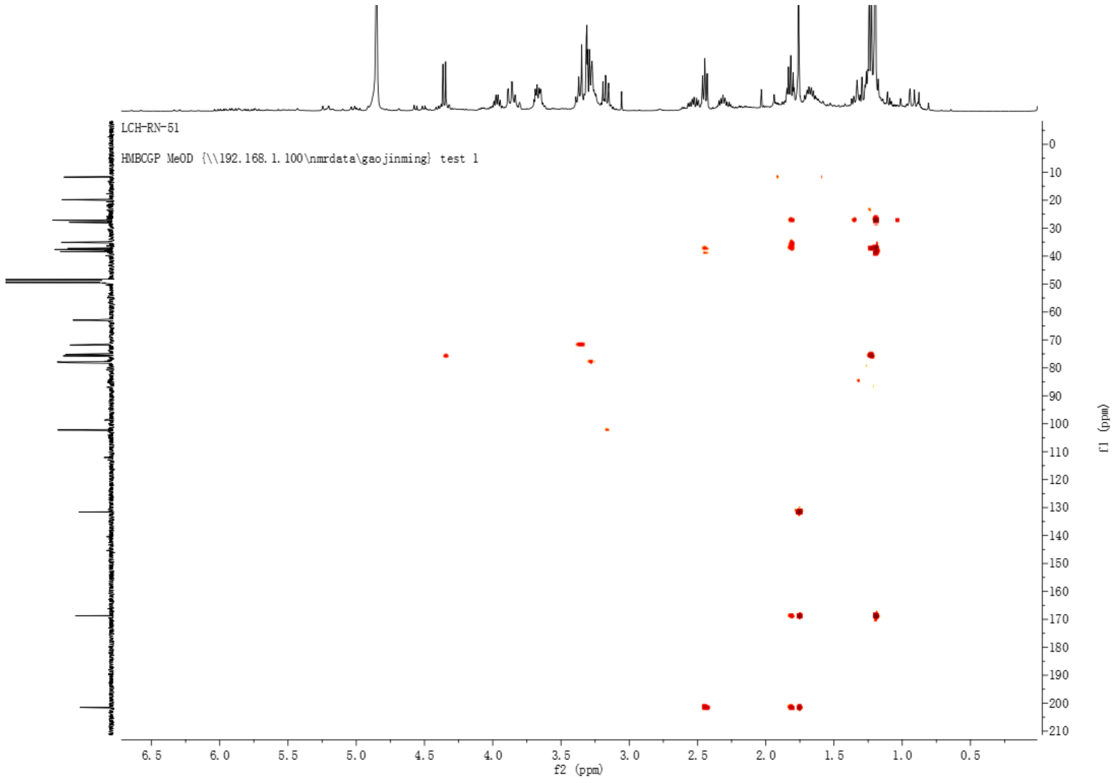

Figure S25. HMBC (400 MHz) spectrum of compound **3** in methanol-d_4

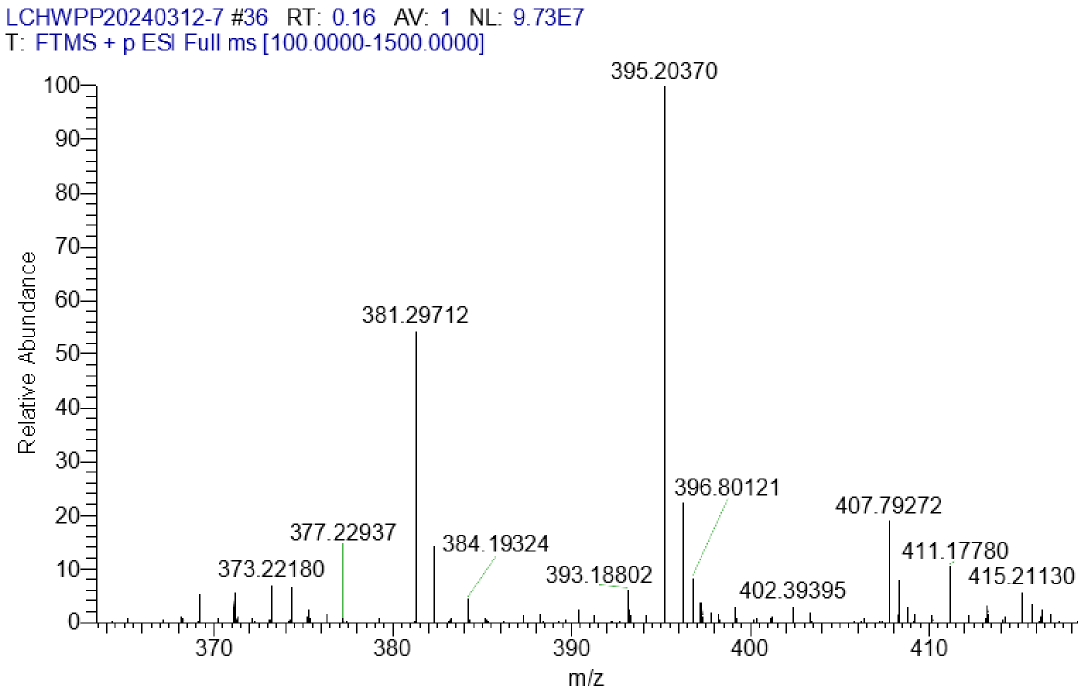

Figure S26. HRESIMS sof compound **3**

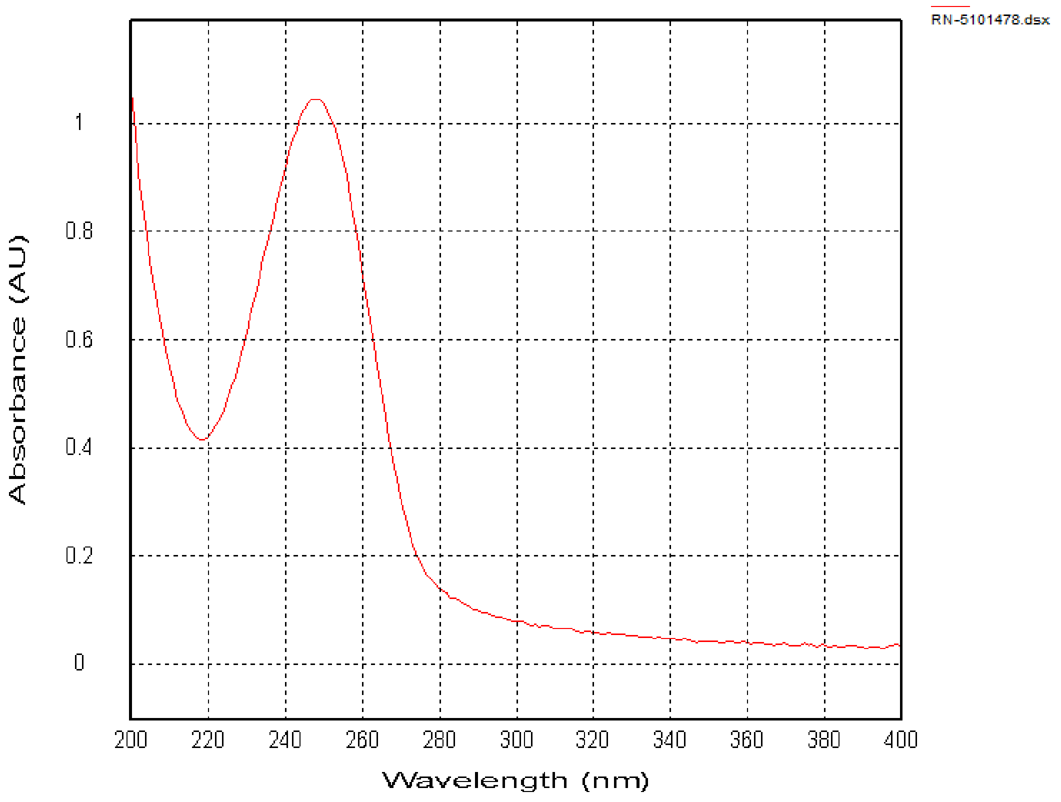

Figure S27. UV spectrum of compound **3** (MeOH)

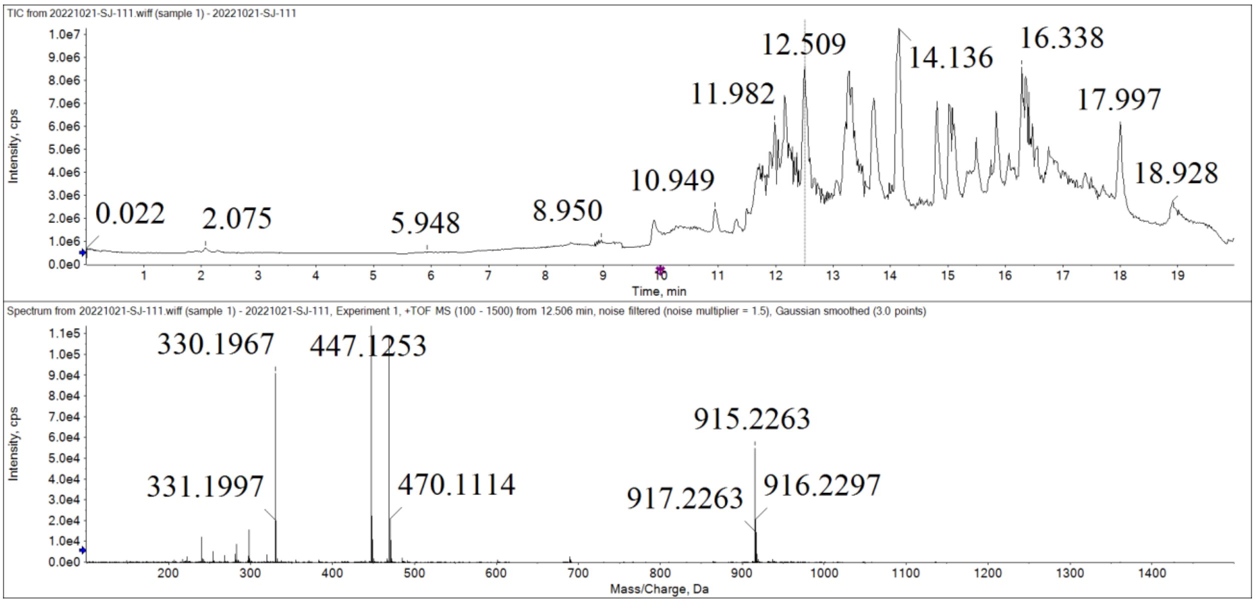

Figure S28. LC-MS spectrum of the glucose moiety of compound **3**

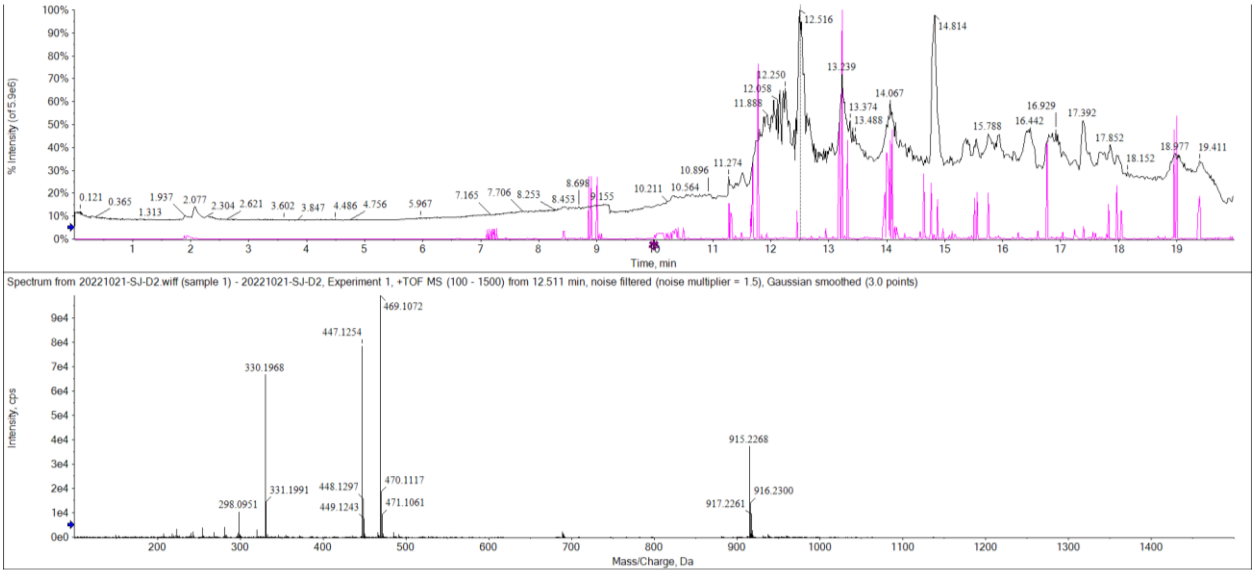

Figure S29. LC-MS spectrum of the D-glucose
